# Development of an Adaptive, Economical, and Easy-to-Use SP3-TMT Automated Sample Preparation Workflow for Quantitative Proteomics

**DOI:** 10.1021/acs.jproteome.5c00124

**Published:** 2025-05-27

**Authors:** Jake N. Hermanson, Lea A. Barny, Lars Plate

**Affiliations:** 1 Department of Biological Sciences, 5718 Vanderbilt University, Nashville, Tennessee 37240-0002, United States; 2 Program in Chemical and Physical Biology, 5718 Vanderbilt University, Nashville, Tennessee 37240-0002, United States; 3 Department of Chemistry, 5718 Vanderbilt University, Nashville, Tennessee 37240-0002, United States; 4 Department of Pathology, Microbiology and Immunology, Vanderbilt University Medical Center, Nashville, Tennessee 37232, United States

**Keywords:** automation, robotics, high throughput, sample preparation, proteomics, tandem mass tag, unfolded protein response, mass spectrometry, bottom-up approach, Biomek i5

## Abstract

Liquid handling robots have been developed to automate various steps of the bottom-up proteomics workflow, however, protocols for the generation of isobarically labeled peptides remain limited. Existing methods often require costly specialty devices and are constrained by fixed workflows. To address this, we developed a cost-effective, flexible, automated sample preparation protocol for TMT-labeled peptides using the Biomek i5 liquid handler (Beckman Coulter). Our approach leverages single-pot solid-phase-enhanced sample preparation with paramagnetic beads to streamline protein cleanup and digestion. The protocol also allows for adjustment of trypsin concentration and peptide-to-TMT ratio to increase throughput and reduce costs, respectively. We compared our automated and manual 18-plex TMT-Pro labeling workflows by monitoring select protein markers of the unfolded protein response in pharmacologically activatable, engineered cell lines. Overall, the automated protocol demonstrated equivalent performance in peptide and protein identifications, digestion and labeling efficiency, and an enhancement in the dynamic range of TMT quantifications. Compared to the manual method, the Biomek protocol significantly reduces hands-on time and minimizes sample handling errors. The 96-well format additionally allows for the number of TMT reactions to be scaled up quickly without a significant increase in user interaction. Our optimized automated workflow enhances throughput, reproducibility, and cost-effectiveness, making it a valuable tool for high-throughput proteomics studies.

## Graphical abstract

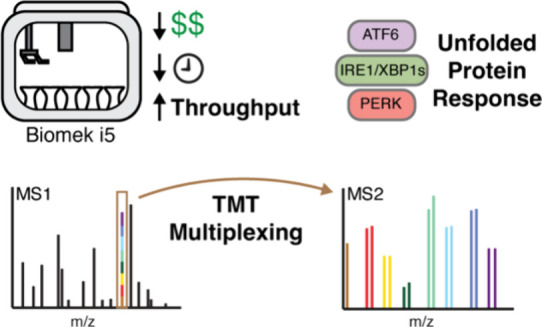

## Introduction

Advancements in mass spectrometry (MS) instrumentation has enabled the study of proteins with high sensitivity and reproducibility in both basic and translational research at an unprecedented scale.^1–6^ Regardless of the application, reproducible sample preparation is essential for minimizing technical variability to avoid obscuring biological insight.^7^ As MS analysis speed increases, sample preparation has become a bottleneck for throughput. To address this challenge, several laboratories have developed protocols using liquid handling robots from various venders: Agilent,^8^ ThermoFisher,^9–11^ Beckman Coulter,^12–15^ Opentrons,^16^ Hamilton Vantage,^17^ and others^10,18,19^ to automate key steps in the bottom-up proteomics workflow—including reduction, alkylation, cleanup, digestion, and acidification, following initial user interaction.^20^ While numerous automated liquid handling protocols have been developed for label free peptide generation, few protocols have been developed for the generation of isobarically labeled peptides.^11,13,16,17^ Isobaric labeling using commercial tandem mass tags (TMT) allows for multiplexing of up to 35 samples for concurrent measurement in a single MS analysis, significantly increasing analysis throughput.^21–23^

Previous methods for the preparation of TMT-labeled peptide samples on automated liquid handling systems have required specialty devices such as a vacuum manifold and positive-pressure apparatus to automate solid phase extraction based cleanup steps.^13^ However, these additions result in higher overall instrument cost and may not be widely applicable to other automated protocols utilized by the laboratory. In contrast, instruments like the Accelerome (ThermoFisher Scientific) have been specifically developed for label-free and isobaric-tagged bottom-up proteomics sample preparations. However, this instrument can only perform preprogrammed workflows and the protocol requires purchasing a commercial kit limited to the preparation of 32 samples per cycle.^11^ While previous studies have utilized Single-Pot Solid-Phase-Enhanced Sample Preparation (SP3)^24^ for protein cleanup and digestion in automated proteomics workflows,^16,17^ none have specifically implemented the SP3 protocol on a Biomek instrument. Compared to Opentrons OT-2 instrument, the Biomek i5 offers a larger deck capacity, enabling more complex workflows across multiple disciplines—including genomics (e.g., RNA/DNA extractions^25^), cell biology (e.g., cell culture^26^), and drug discovery—with minimal manual intervention. Additionally, its 96-channel multichannel head accelerates reagent additions, enhancing efficiency and throughput.

To address the need for additional flexible and cost-effective automated sample preparation methods for TMT labeled peptides, we developed an automated workflow on a Biomek i5 liquid handler (Beckman Coulter). Our method begins with unnormalized cell lysate and produces either 16- or 18-plex TMT-labeled samples ready for MS analysis (Figure 1A). The protocol leverages commercially available SP3 beads with specialized surface chemistries for protein binding.^27^ The paramagnetic property of these beads make them particularly conducive to automation and wash steps needed to remove detergents, buffers, etc.^8^ Additionally, our automation protocol allows for the adjustment of trypsin concentration to shorten the digestion incubation^28^ and variation of peptide-to-TMT concentration to increase the number of labeling experiments that can be performed with a set of purchased TMT reagents.^29^ Utilizing our 96-well automation format in combination with TMT isobaric tags enables the parallel preparation of six 16-plex or four 18-plex multiplexed MS pools at a rate of 10.8 min per sample (TMT channel) which is less than the Accelerome at 12.6 min per sample (Figure 1 **B,** Table S1).

**Figure 1:**
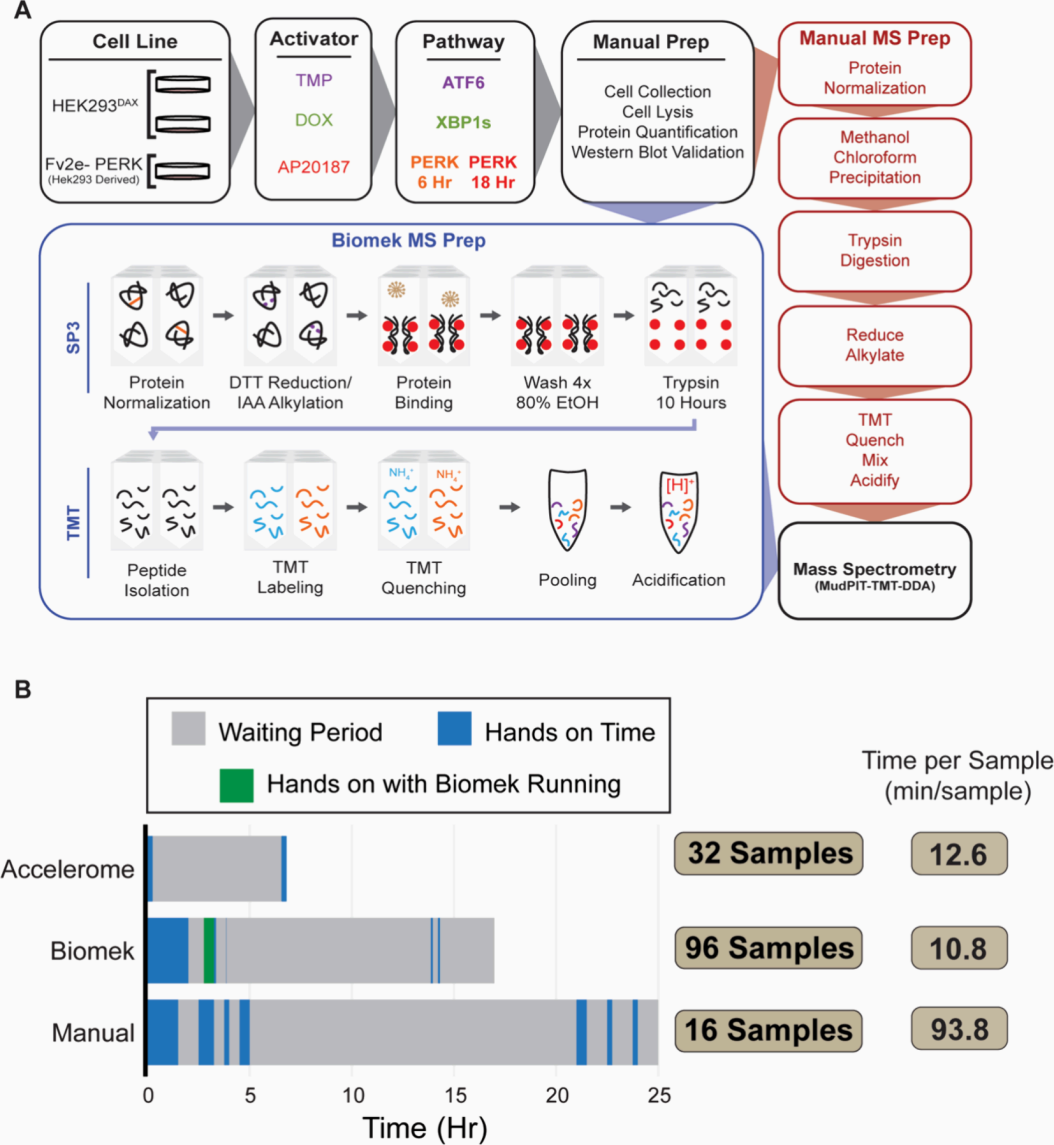
Overview of the Biomek i5 and manual TMT-labeling methods and comparison of the time required to carry out the protocols discussed. (A) Outline of the Biomek and manual preparation method used in this study for the generation of TMT-labeled peptides. The Biomek automated method used SP3 beads for protein cleanup and digestion, while the manual protocol employed a Methanol/Chloroform precipitation technique. (B) Comparison of the time (hr) required to prepare TMT labeled peptides using either the Accelerome (ThermoFisher Scientific), Biomek i5 (Beckman Coulter), or our manual method. Hands-on time includes preparing reagents (both on Biomek and manually) and, for the Biomek specifically, loading samples onto the deck and setting up pipet tips.

We validated our automated workflow by determining activation of the Unfolded Protein Response (UPR), a biologically important pathway with implications in diabetes,^30,31^ neurodegeneration,^32–34^ and cancer.^35–37^ The UPR is triggered by the accumulation of misfolded and aggregated proteins in the endoplasmic reticulum (ER). This response involves three distinct pathways: ATF6, IRE1/XBP1s, and PERK (Figure 2).^38–41^ Activation of each branch of the UPR results in a distinct transcriptional and translational response^41–44^ in which pathway-specific genes and proteins are subsequently upregulated. Branch-specific protein markers can be monitored via liquid chromatography-MS/MS (LC-MS/MS) to define the state of the repsonse.^45,46^ To elicit the UPR response branch-specifically, engineered stable cell lines which can be activated with small molecules were utilized.^42^ We then prepared TMT-Pro labeled 18-plex samples using the automated pipeline and a conventional manual protocol.^47–49^ The manual and automated workflow showed a similar number of peptides and proteins identified with equivalent precision across treatments. Additionally, similar tryptic digestion, alkylation, and TMT labeling efficiencies were observed between the methods. The Biomek preparation method resulted in higher abundances of many core UPR target proteins, highlighting an enhanced quantification dynamic range, while the coefficient of variation (CV) for these proteins remained consistent between the methods. Lastly, compared to the manual TMT label preparation, our automated workflow significantly decreased hands-on time from 24 to 2.8 h, respectively, for the generation of 96 samples. The reduction in hands-on time minimizes the risk of sample mix-ups and reagent addition errors. Implementing our developed workflow will enhance the throughput of samples processed for MS analysis in a reproducible and cost-effective manner.

**Figure 2:**
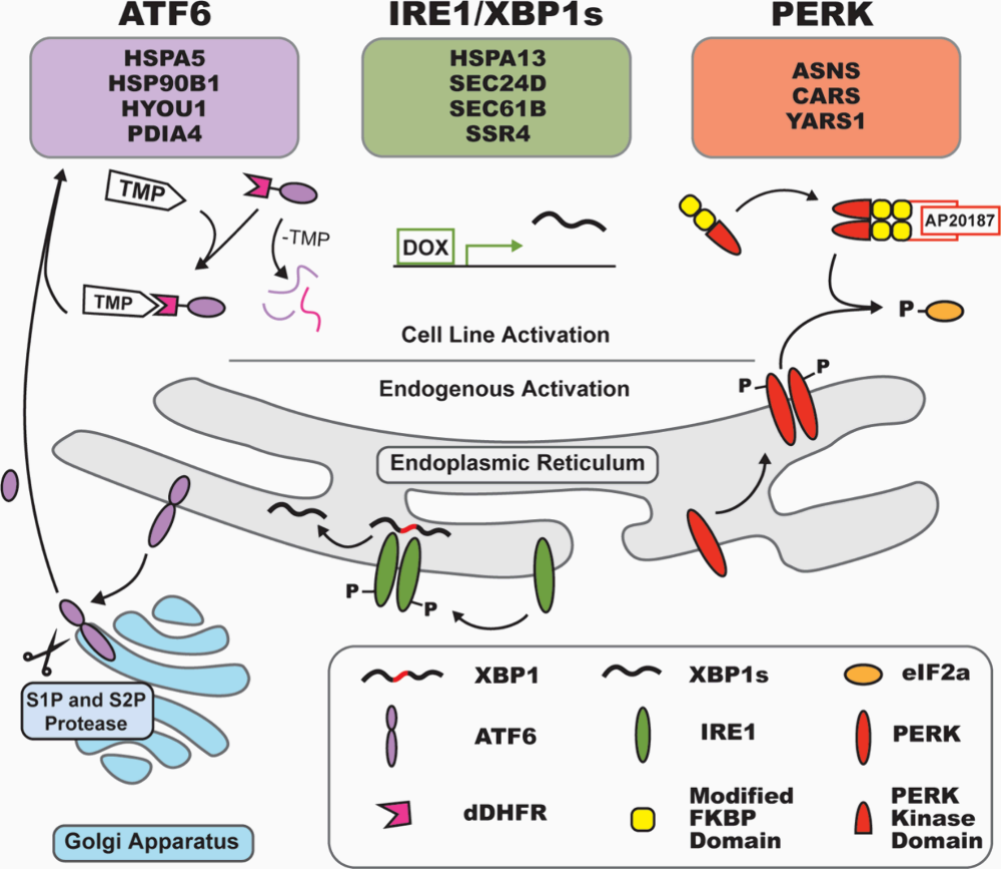
Overview of the three branches of the Unfolded Protein Response (UPR). Known branch-specific UPR target protein markers were used throughout this study to monitor the state of the response. At the top of the figure, several genes are highlighted that are upregulated during activation. ATF6 activation causes upregulation of HSPA5, HSP90B1, HYOU1, and PDIA4. HSPA13, SEC24D, Sec61B, and SSR4 are upregulated by XBP1s, while ASNS, CARS, and YARS1 are upregulated upon PERK activation. Endogenous activation of ATF6 occurs when ATF6 translocates from the endoplasmic reticulum to the golgi apparatus. Cleavage by S1P and S2P proteases occur here to release ATF6-N. This truncated form of ATF6 then moves to the nucleus and acts as a transcription factor. In the HEK293^DAX^ engineered stable cell line, the active ATF6-N is tagged with destabilized DHFR (dDHFR). This tag targets ATF6-N for degradation, but when treated with TMP stabilizes dDHFR, allowing for ATF6-N to act as a transcription factor. IRE1/XBP1s is activated when IRE1 autophosphorylates and noncanonically splices the XBP1 mRNA into XBP1s which is translated into a transcription factor. In the HEK293^DAX^ cells, a doxycycline (dox)-inducible Tet-On system is used where XBP1s is transcribed in the nucleus and does not require additional splicing. Lastly, endogenous PERK becomes active when it dimerizes, autophosphorylates and in turn phosphorylates eIF2α. Activation of the Fv2e-PERK cell line occurs with a fusion protein containing the PERK kinase domain and two FKBP domains that when treated with AP20187 cause dimerization and subsequent activation. The ATF6, XBP1s, and PERK branches of the UPR were pharmacology activated with TMP (10 μM), DOX (1 μg/mL), and AP20187 (5 nM) for 16–18 h, respectively.

## Materials and Methods

### Cell Culture and Harvesting

HEK293^DAX^ and HEK293 Fv2e-PERK cells were previously established as engineered stable cell lines to study the UPR and were a kind gift from Luke Wiseman (Scripps Research Institute).^42,55^ Cells were cultured in Dulbecco’s modified Eagle medium supplemented with 10% fetal bovine serum, 1% penicillin/streptomycin and 1% glutamine (Incubated at 37 °C with 5% CO_2_). To induce the upregulation of ATF6 or XBP1s transcription factors in the HEK293^DAX^ cell line, cells were incubated with either 10 μM TMP or 1 μg/mL doxycycline (DOX), respectively, for 18 h. To promote PERK transmembrane receptor dimerization, Fv2e-PERK cells were treated with 5 nM of AP20187 and were harvested at both 6 and 18 h post drug addition. Cells were washed 2× with ice cold phosphate buffered saline (PBS), then scraped and collected in ice cold PBS + 1 mM ethylenediaminetetraacetic acid (EDTA), followed by cell pelleting (200 g, 5 min). The pellet was washed 2× with cold PBS and centrifuged at 200 g for 5 min. Remaining PBS was removed by suctioning followed by addition of radioimmunoprecipitation assay (RIPA) buffer (50 mM Tris (pH 7.5), 150 mM NaCl, 0.1% SDS, 1% Triton X-100, 0.5% deoxycholate) containing 1× Protease inhibitor (Roche complete EDTA-free protease inhibitor Millipore Sigma, 4693132001; HEK293^DAX^ cells) and 1× Protease +0.1% (v/v) phosphatase inhibitor (Fv2e-PERK cells) for 15 min. Insoluble debris was then pelleted via centrifugation (16k × g, 15 min) followed by quantification of protein levels via bicinchoninic acid assay.^53^

### Automated Sample Preparation

The method for the Biomek i5 system can be found in the supplement. Cell lysate was normalized to 20 μg of protein in 70 μL RIPA buffer on the Biomek i5 followed by SP3 bead digestion and cleanup described elsewhere.^24^ Briefly, proteins were first reduced with 5 mM DTT at 60 °C/700 rpm for 30 min then alkylated with 20 mM IAA for 30 min at RT in the dark. Finally, the reaction was quenched with a second addition of 5 mM DTT for 15 min at RT. Proteins were digested on 8 μL total of SP3 beads (Cytiva; 1:1 hydrophilic to hydrophobic beads; 50 mg/mL) in 50 μL of 50 mM HEPES (pH 8) with Trypsin/Lys-C in a 1:40 protease to protein ratio for 10 h at 37 °C/700 rpm. After digestion, peptides were held at 4 °C until TMTPro labeling. To begin the labeling protocol, peptides were first transferred to a new PCR plate (free of SP3 beads) followed by the addition of 10 μL H_2_O. Peptides were then reacted with 40 μL of resuspended TMTPro 18-plex reagent for 1 h at room temperature. Purchased TMTPro reagent was dissolved in acetonitrile (ACN) to achieve a final concentration of 2.42 mg/mL. For ease of use on the automated protocol, TMT reagents were stored in MX500 LVL tubes (Thomas Scientific). The TMT reaction was subsequently quenched with the addition of fresh 10% ammonium bicarbonate (w/v in H_2_O) to a final concentration of 4% (v/v) and incubated for 1 h at room temperature. Samples were then pooled into a fresh LoBind tube (ThermoFisher Scientific) and acidified using formic acid (FA, ThermoFisher Scientific, PI28905) to ensure pH ≤ 2.0. The pooled sample was then dried *in vacuo* using a SpeedVac (Thermo Fisher) until a third of the volume total (after labeled peptide pooling/acidification) remained. The volume of the sample was then adjusted back to the original pooled volume with buffer A (95% H2O, 4.9% ACN, 0.1% FA), centrifuged for 30 min at 21.1k × g to remove residual beads, and the supernatant was transferred to a fresh LoBind tube prior to LC-MS/MS analysis.

### Manual Sample Preparation

Cell lysate was normalized to 20 μg of protein in 100 μL RIPA buffer followed by methanol/chloroform precipitation. Protein was precipitated by adding methanol (MeOH), chloroform, and water in a 3:1:3 ratio, respectively. The sample was then vortexed and spun down for 1 min at 21.1k × g, followed by removal of the top layer and two additional washes with 500 μL MeOH. After the final wash, MeOH was suctioned off and samples were then left to air-dry. Protein pellets were then resuspended in 5 uL 1% RapiGest SF (w/v; Waters #186002122) surfactant followed by an addition of 10 uL of 0.5 M HEPES (pH 8.0). Volume was then adjusted to 47.5 uL with H_2_O. Reduction of proteins was carried out with 5 mM TCEP (30 min at RT) followed by protein alkylation (30 min, dark at RT) using 10 mM iodoacetamide. Next, proteins were digested overnight (10 h) at 37 °C using Trypsin/Lys-C at a 1:40 protease to protein ratio while shaking at 750 rpm. Samples were TMT labeled in the same manner as described in the Automated Sample Preparation section. Lastly, samples were pooled and acidified with FA (added to reach pH 2). The sample was then concentrated on the SpeedVac to one-third of the pooled volume to remove ACN. The volume of the sample was then adjusted back to the original pooled volume with buffer A, centrifuged for 30 min at 21.1k × g to remove RapiGest SF, and the supernatant was transferred to a fresh lobind tube prior to LC-MS/MS analysis.

### Liquid Chromatography-Tandem Mass Spectrometry

LC-MS/MS data-dependent analysis was performed using an Exploris 480 mass spectrometer (Thermo Fisher) equipped with a Dionex Ultimate 3000 RSLCnano system (Thermo Fisher). MudPIT columns were made as previously described^59^ with 15 μg of labeled peptides loaded onto the trap column using a high pressure chamber and washed for 30 min with Buffer A prior to MS analysis. To elute peptides from the first C18 phase to the SCX resin of the MudPIT column, 10 μL of Buffer A was injected and the following 90 min gradient was utilized: 2% B (5 min hold) ramped to a mobile phase concentration of 40% B over 35 min, ramped to 80% B over 15 min, held at 80% B for 5 min, then returned to 2% B in 5 min and then held at 2% B for the remainder of the analysis at a constant flow rate 500 nL/min. The sample was fractionated into 11 fractions through sequential 10 μL injections of buffer C (500 mM ammonium acetate in buffer A) in Buffer A, increasing in 10% increments from 10% to 100%. A final fraction was collected using 90% buffer C and 10% buffer B (99.9% acetonitrile, 0.1% formic acid v/v). Each fraction was separated on a 130 min gradient at a flow rate of 500 nL/min (4% B for 10 min then ramped to 40% B over 90 min, increased to 80% B over 5 min then held at 80% B for 5 min and returned to 4% in 5 min, and held at 4% for the remainder of the analysis). For TMT-DDA acquisition, a 3 s duty cycle was utilized consisting of a full scan (400–1600 *m*/*z*, 120,000 resolution) and subsequent MS/MS spectra collected in TopSpeed acquisition mode. For MS^1^ scans, the maximum injection time was set to 50 ms with a normalized AGC target of 100%. Ions were selected for MS/MS fragmentation based on the following criteria: MS^1^ intensity above 1e4, charge state between 2 and 6, and monoisotopic peak determination set to peptide. Additionally, a dynamic exclusion time of 45 s was utilized (determined from peptide elution profiles) with a mass tolerance of ± 10 ppm to maximize peptide identifications. MS/MS spectra were collected with a normalized HCD collision energy of 36, 0.4 *m*/*z* isolation window, auto selected for maximum injection time mode, a normalized AGC target of 100%, at a MS^2^ orbitrap resolution of 45,000 with a defined first mass of 110 *m*/*z* to ensure measurement of TMTPro reporter ions.

### Data Analysis

Proteome Discoverer 2.4 (Thermo Fisher Scientific) was utilized to obtain peptide identifications and TMT-based protein quantification. MS/MS spectra were searched using SEQUEST-HT against a Uniprot SwissProt canonical human FASTA database (downloaded 01May2024; containing 20,361 entries), contaminant FASTA^60^ (containing 379 entries), and a decoy database of reversed peptide sequences. Peptide precursor ion mass tolerance was set to 20 ppm, and fragment ion mass tolerance was fixed at 0.02 Da. Only peptides with a minimum length of six amino acids were considered, and trypsin digestion was assumed with a maximum of two missed cleavages allowed. Dynamic modifications included oxidation of methionine (+15.995 Da), protein N-terminal methionine loss (−131.040 Da), protein N-terminal acetylation (+42.011 Da), and a combination of N-terminal methionine loss and acetylation (+89.030 Da). Static modifications included cysteine carbamidomethylation (+57.021 Da) and TMTpro labeling (+304.207 Da) on the N-terminus and lysine. To determine TMT labeling and alkylation efficiencies, the TMTpro and carbamidomethylation modifications were set to dynamic instead of static in Proteome Discover. The proportion of modified vs unmodified PSMs were then determined.

To control false discoveries, the peptide-level FDR was set to 1% using the Percolator algorithm. Quantification of TMT reporter ions was performed by including only those with an average signal-to-noise ratio greater than 10:1 and a coisolation percentage of less than 25%. TMT reporter ion intensities were summed for peptides assigned to the same protein, including razor peptides, which are shared by multiple proteins but assigned to the one providing the most confident identification. Protein identifications were filtered at a 1% FDR threshold, and protein grouping was conducted according to the parsimony principle, which minimizes the number of protein groups assigned to each peptide.

Median normalization was subsequently carried out using custom R code available on GitHub (https://github.com/Plate-Lab/). Briefly, correction factors per TMT channel were applied to the raw abundances of each observation in the corresponding channel. The mass spectrometry proteomics data are deposited to the ProteomeXchange Consortium via the PRIDE partner repository under the accession code PXD060786. All other necessary data are contained within the manuscript or can be shared by the Lead Contact upon request.

## Results and Discussion

### Establishing an Instrument Workflow on the Biomek i5 for the Generation of TMT-Labeled Peptides

The Biomek i5 is a versatile liquid handler capable of automating sample preparation for a variety of applications, including genomic analysis (qPCR, transcriptomics, etc.)^25,50^ and LC-MS/MS workflows, particularly for proteomics.^13,14,51,52^ Here, we present an optimized protocol using this instrument to prepare 96 TMT labeled peptide samples ready for mass spectrometry (Figure 1A). Briefly, the protocol begins with lysate normalization following off-line determination of protein concentration in whole-cell lysates using a bicinchoninic acid assay.^53^ A CSV file containing the buffer and lysate amounts for normalization to 20 μg of protein is then uploaded to the instrument for automated normalization into a deep-96 well plate. Proteins are then reduced and alkylated using dithiothreitol (DTT) and iodoacetamide (IAA), respectively, and subsequently bound to SP3 paramagnetic beads for cleanup prior to digestion with Trypsin/Lys-C. For steps requiring temperature control and/or shaking, the Bioshake Q1 Heater Cooler Shaker (QINSTRUMENTS) was used. For example, during normalization and after protein digestion samples were held at 4 °C until further user interaction. Samples can then either be collected for label-free mass spectrometry or remain on the Biomek i5 for TMT labeling, quenching, pooling, and acidification.

In contrast, the manual preparation involved normalizing samples by hand and performing methanol/chloroform precipitation for protein isolation. Briefly, proteins were resolubilized in 1% RapiGest and reduced with tris­(2-carboxyethyl)­phosphine (TCEP), followed by alkylation with IAA. All other steps in the TMT-labeled peptide generation process mirrored the Biomek i5 protocol but were executed manually instead. Initial iterations of this protocol involved the movement of RapiGest resuspended proteins onto the Biomek following methanol/chloroform precipitation for subsequent reduction, alkylation, digestion and TMT labeling. While this method resulted in high peptide and protein identifications, transfer of protein in this manner was extremely time-consuming and risked protein loss, prompting the need to optimize an automated SP3 method. Magnetic bead-based workflows offer the advantage of scalability, allowing for the processing of low to high protein amounts by adjusting the bead quantity. SP3 beads, in particular, are well-suited for low-input samples, accommodating as few as 100 cells and inputs as low as 100 ng.^8,54^ Our current protocol accommodates an initial starting volume of 70 μL in a buffer compatible with SP3 preparation, allowing flexibility in protein digestion amounts. Proper adjustment of SP3 bead input based on their binding capacity (10:1, bead-to-protein ratio by weight) ensures efficient peptide recovery.^24^ Following digestion, paramagnetic SP3 beads are easily precipitated from peptides using a removable universal magnet plate without the need for further peptide cleanup steps.

Following digestion, TMT-Pro labeling of peptides was carried out either manually or on the Biomek. To facilitate easier storage and dispensing on the Biomek, the TMT reagents were stored in LVL tubes. LVL tubes are individual, barcoded tubes with screw caps that are available for purchase in a 96-well format. These tubes can be filled with variable quantities of TMT reagent, providing flexibility in reagent storage and dispensing. While this method is convenient, it is optional. Alternatively, TMT reagents can be manually transferred to a 96-deep-well plate or purchased prepackaged in a 96-well format. Our instrument is additionally outfitted with a High Efficiency Particulate Air (HEPA) filtration system to prevent sample contamination from air particles. Overall, the only specialized devices required to run our protocol are the Bioshake Q1 Heater Cooler Shaker and removable magnetic plate.

A key consideration when utilizing liquid handling systems is the requirement for additional reagent volume to ensure accurate aspiration. The estimated reagent cost for a full 96-well plate TMT 16-plex preparation on the Biomek, including dead volume, is approximately $1,308 (based on a 5 μg TMT 16-plex kit). Consumables cost approximately $70 per run, excluding pipet tips, which contribute an additional $150. Altogether, the total cost per TMT 16-plex is approximately $255 when 96 samples (six 16-plexes) are processed. An additional benefit of utilizing low TMT labeling stoichiometry is that approximately 50 TMT 16-plexes can be labeled per 5 μg purchased TMT reagent set.

One key difference between the methods described is the time required to generate a single TMT-labeled sample. The entire Biomek and manual protocol generates TMT labeled samples at a rate of 10.8 and 93.6 min/sample, respectively (Figure 1B). In addition to passive incubations in which the user is not required to engage with the instrument or method, indicated as a “waiting period” in Figure 1B in gray, the Biomek protocol also requires manual reagent preparation (bead washing, trypsin, etc.) while the instrument is performing reduction and alkylation steps, indicated in green as “hands on with Biomek running” (Figure 1B). The ability to add reagent to all wells in use on the Biomek significantly decreases the manual time required for sample preparation. Overall, the Biomek-assisted protocol requires approximately 2.8 h of manual intervention to generate 96 TMT-labeled samples, compared to 24 h for the fully manual protocol. Alternatively, the Accelerome (ThermoFisher Scientific) generates samples at a rate of 12.6 min/sample. The sample production rate is reduced using the Accelerome compared to the Biomek largely due to the number of samples that can be prepared at a given time (Figure 1B). Additionally, the shortened processing time enabled by the Accelerome platform is achieved by using a higher concentration of trypsin, which facilitates faster digestion of the lysate.

### Comparing the Performance of the Preparation Techniques: Manual vs Biomek i5

To validate our developed Biomek protocol, we utilized established engineered stable cell lines (HEK293^DAX^ and Fv2e-PERK) in which the three branches of the UPR can be activated individually with small molecules (trimethoprim (TMP), doxycycline (DOX), AP20187 (cell-permeable dimerizer of FK506-Binding Protein (FKBP) fused proteins)), allowing us to test the differences between the manual and Biomek i5 preparation protocols (Figure 2).^42,55^ We employed well-characterized antibody-based markers for branch-specific UPR activation, such as BiP for ATF6 activation and eIF2α-P for early PERK activation (6 h), followed by ASNS upregulation for late PERK activation (18 h). UPR activation in cell lines was confirmed by Western blotting prior to MS analysis (Figure S1)

Both manual and Biomek prepared TMT 18-plex samples were analyzed using Multidimensional Protein Identification Technology (MudPIT) – data dependent acquisition (DDA).^56^ MudPIT utilizes ion exchange chromatography to fractionate complex peptide mixtures, enhancing peptide detection.^57–59^ For each analysis, identical amounts of peptides were loaded on-column. To determine the number of peptide groups and proteins identified using the two sample preparation protocols, we first removed known common contaminants^60^ and retained proteins with a minimum of two unique peptides. Using these criteria, 24,061 peptide groups (e.g., different forms of the same peptide, such as distinct charge states or variable modifications) were identified with manual preparation, while 24,052 peptide groups were identified with the Biomek i5 preparation (Figure 3A). Alternatively, 3,544 and 3,676 proteins were identified using the manual and Biomek i5 automated preparation, respectively (Figure 3 **B,** Table S2 & S3). High overlap was observed between the methods in identified peptide groups and proteins (Figure 3 **C/D**).

**Figure 3:**
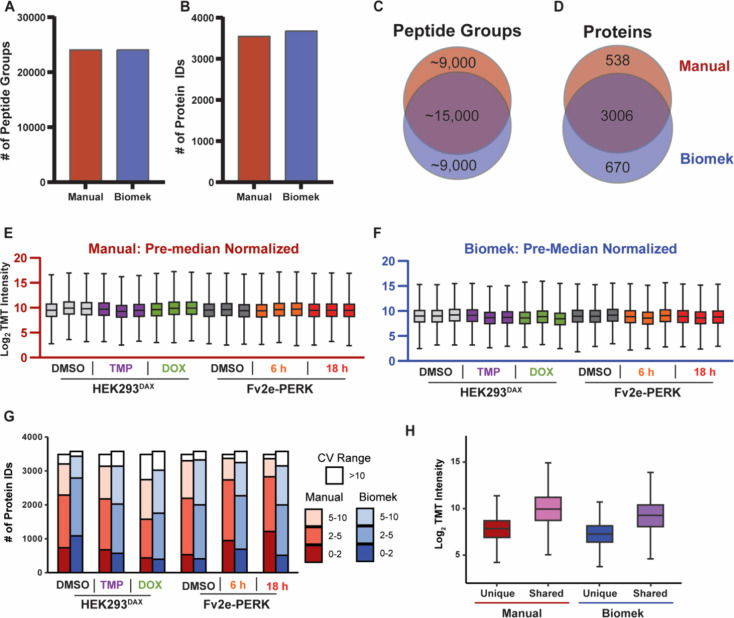
Biomek and manual sample preparation show similar performance in peptide and protein identifications. (A, B) Bar graphs indicate the number of peptide groups and proteins detected, in all TMT channels, using either the manual or Biomek i5 preparation. (C, D) Unique and shared peptide groups and proteins identified between the two methods. The manual preparation had ∼24,000 peptide groups and 3,544 protein IDs in total while the Biomek method had ∼24,000 peptide groups and 3,676 protein IDs. (E, F) Distribution of protein abundances pre median normalization. (G) Coefficient of variation (CV %) ranges of the proteins identified in each of the cell lines with activation, comparing the TMT sample preparation methods. (H) Comparison of protein abundances between uniquely identified proteins in each run and proteins detected in both runs. Proteins identified in both runs reflect consistently detectable proteins and illustrate overall abundance within each data set. Box and whisker plots represent all peptide TMT intensities, separated by treatment. The top and bottom of the box represent the third (Q3) and first (Q1) quartiles, respectively, with the middle line indicating the median. The whiskers extend to the smallest and largest values within 1.5 times the interquartile range (IQR).

To compare the precision of the workflows, we assessed the median abundances of each sample before and after median normalization, along with the distribution of protein CVs. When comparing the distribution of protein abundances prior to normalization, neither preparation had samples that deviated far from the average median, indicating the methods did not result in high sample loss (Figure 3 **E/F**). After median normalization, all samples had a similar median abundance, regardless of the sample preparation technique utilized (Figure S2A/B). Lastly, CVs were calculated for all identified proteins on the nonlog-transformed intensities of biological replicates, grouped by treatment and cell line, after applying global median normalization using the equation CV= σ (mean)/ μ (standard deviation).^61^ The number of proteins with a CV less than 10 (indicating high precision) was similar between the methods, however the Biomek preparation had only slightly more proteins that were identified with CVs greater than 10 (9.9% vs 8.6%) (Figure 3G). Furthermore, to investigate the source of unique protein identifications between sample preparations, we compared the intensities of proteins identified in both runs with those uniquely identified in each run (Figure 3H). Our analysis revealed that proteins unique to specific sample preparation methods exhibited lower abundances, suggesting that their detectability is more variable in DDA workflows. (Figure 3H). Overall, these methods show similar precision in their preparations.

### Comparing Efficiency of Trypsin Digestion, Alkylation, and TMT Labeling

To better understand the potential differences in sample preparation between the manual and automated protocols, we assessed the efficiencies of digestion, alkylation, and TMT labeling. First, to evaluate trypsin digestion efficiency, we compared the number of missed cleavages between the two TMT peptide preparation protocols. Both protocols resulted in a similar number of missed cleavages, indicating that the digestion time and trypsin concentration utilized was suitable for the trypsin-to-lysate ratio (Figure 4A). In our protocol, Trypsin/Lys-C was utilized at a 1:40 enzyme/substrate ratio for 10 h. Importantly, higher enzyme concentrations can be utilized to reduce digestion times, allowing for an additional gain in sample preparation throughput.^62,63^ This approach, however, is more costly compared to the enzyme concentrations used in this study.

**Figure 4:**
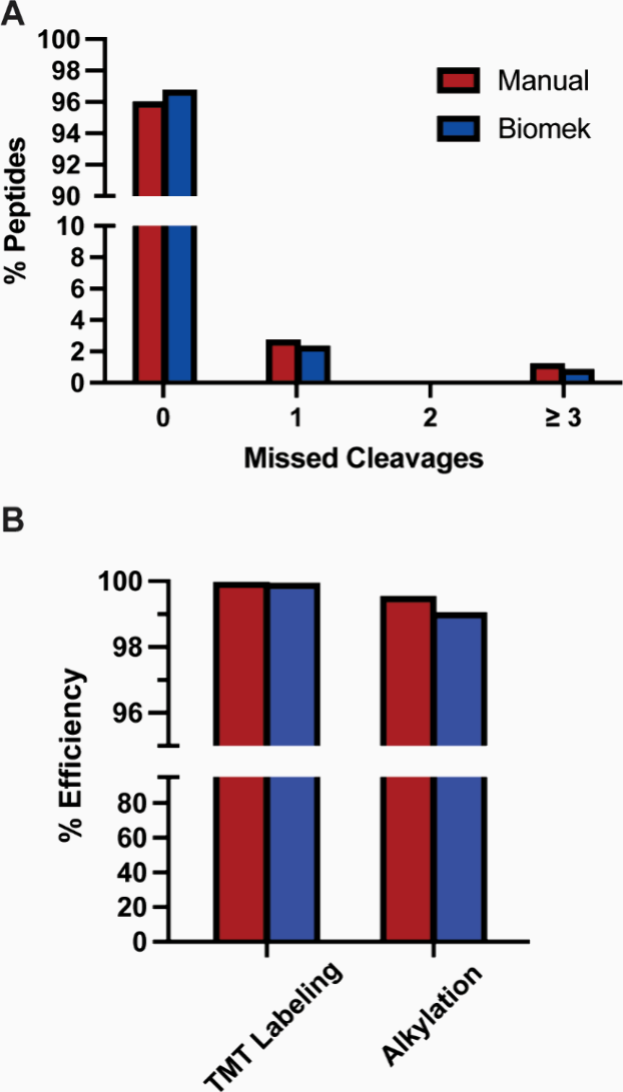
Biomek and manual preparation show comparable labeling and digestion efficiencies. (A) Percentage of peptides with zero, one, two, or more than three Trypsin/Lys-C missed cleavages detected in both manual and Biomek i5 TMT sample preparations. (B) TMT labeling and alkylation efficiency of the two preparations.

Alkylation and TMT labeling efficiencies were evaluated by searching the data with carboxyamidomethylation and TMT-Pro modifications, respectively, as dynamic rather than static variables to determine the number of peptides missing the desired modification. Importantly, the methods did not result in different alkylation or TMT labeling efficiencies (Figure 4B). Both preparations yielded TMT labeling efficiencies over 99%. These data indicate that the quality of sample preparation is consistent, whether performed manually or using the automated protocol. Importantly, our TMT labeling method differs from the manufacturer’s protocol in that the peptide to TMT reagent ratio was 1:5 as opposed to the recommended 1:25. Previously, it was reported that peptide-to-TMT ratios as low as 1:1 (weight/weight) result in labeling efficiencies exceeding 99%.^29^ Our automated TMT labeling approach makes the technique cost-effective and scalable for large-scale studies.

### Evaluating TMT-Based Quantification in the Context of the Unfolded Protein Response (UPR)

To determine whether the automated or manual preparation provided better quantification accuracy the upregulation of known protein markers for each branch of the UPR were assessed.^42,45^ For the majority of branch specific UPR markers evaluated, the log_2_ fold change of the treatment compared to the DMSO control was greater for the Biomek preparation than that of the manual preparation (Figure 5 **A,** Figure S3A/B). This was especially pronounced for the ATF6 branch of the UPR pathway. We thus compared ATF6 activated samples prepared either manually or with the Biomek to reveal higher abundances for many of the prominent upregulated ATF6 markers in the Biomek preparation (Figure 5B). These data suggest that the method of protein cleanup and isolation (e.g., SP3 or Methanol/Chloroform) can influence the recovery of specific proteins, especially those with a high dynamic range, such as those upregulated during the UPR response.^64^ The ATF6-associated proteins, namely HSPA5, HSP90B1, and PDIA4, exhibited the greatest differences in abundance between the two preparation methods in TMP-treated HEK293^DAX^ cells, and were also the most differentially expressed proteins in the data set. Similar trends were observed for proteins regulated by XBP1s and PERK (assessed at 6 h and 18h), although the differences in abundance between the two methods were less pronounced, likely because these proteins are not as strongly differentially expressed (Figure S3C/D).

**Figure 5:**
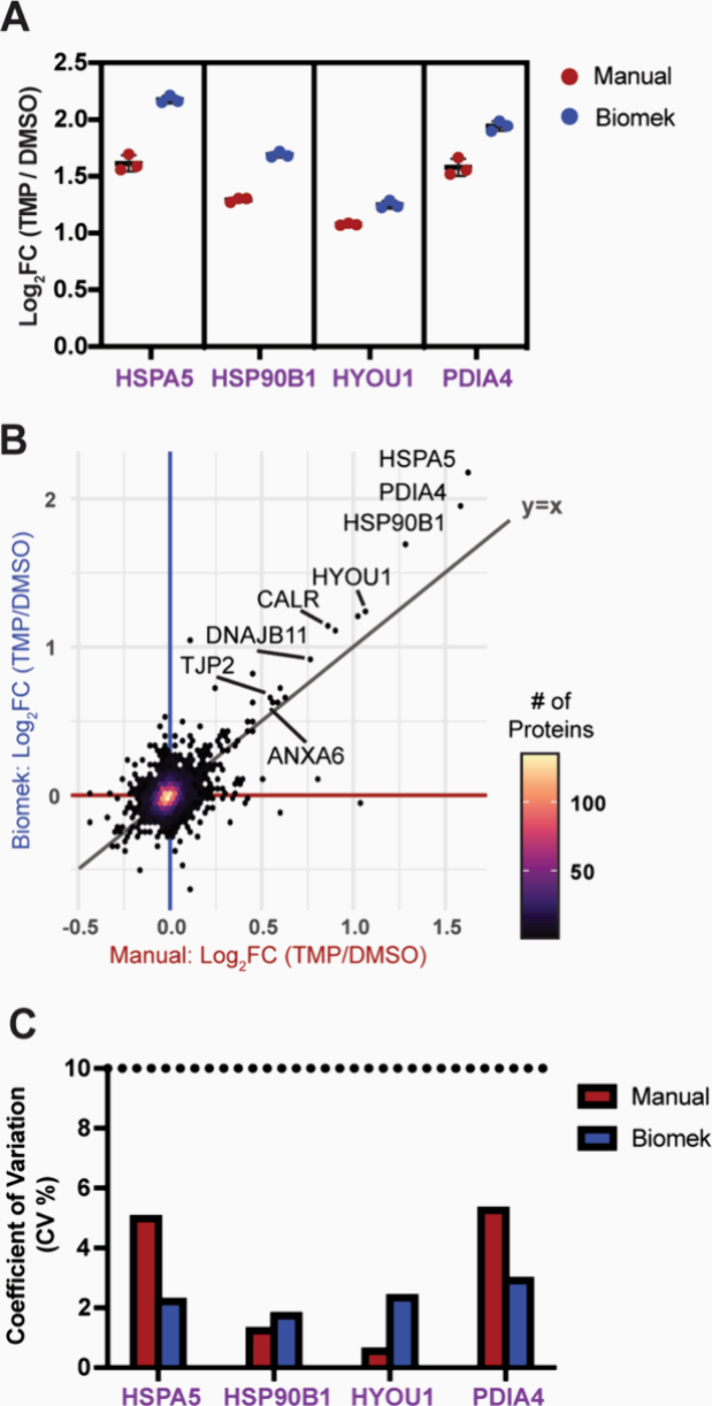
Identification of core UPR protein markers in the Biomek and manual preparations. (A) Log_2_ fold change of core ATF6 target proteins when prepared with the two methods. (B) Correlation plot of the log_2_ fold change of TMP activated HEK293^DAX^ cells compared to DMSO in both preparation methods. (C) Coefficient of variation of select AFT6 regulated UPR target proteins.

Second, the CV of the branch-specific UPR proteins was monitored to assess whether the methods differed in the variation introduced during sample preparation **(** Figure 5 **C)**. Low variability (CV% < 10) was observed within biological replicates of the sample condition, indicating that the methods provide robust sample preparation (Figure S3E/F). Overall, these data reveal the SP3 beads preparation, executed on the Biomek, promotes identification of select UPR markers with high precision. As these markers in different biological contexts may be upregulated to different extents, a technique that is able to accommodate a large dynamic range is preferable.

After confirming that the manual preparation performs comparably to the automated preparation, we proceeded to validate the reproducibility of the automated TMT preparation. To achieve this, we conducted three separate 18-plex TMT experiments on different days, with each experiment containing different biological replicates for each drug treatment. Lysates of the various biological replicates were validated for branch-specific UPR activation prior to Biomek sample preparation. The number of identified peptide groups and proteins was consistent across replicates, ranging from 23,154 to 34,507 peptides and 3,443 to 4,293 proteins, respectively **(** Figure 6 **A/B)**. As observed previously, the number of peptide groups and protein identifications were comparable to the manual preparation (Figure 3 **A/B**). Although some variation between replicates was observed, which could be attributed to either preparation or biological variability, the protein overlap remained high, with 2,697 proteins consistently identified across all replicates **(** Figure 6 **C)**. Additionally, the degree of UPR activation for HSPA5 was similar in replicates 2 and 3 and slightly lower for replicate 1, while PDIA4 showed comparable activation across all replicates **(** Figure 6 **D)**. This suggests that certain biological replicates may have minimally stronger activation due to differences in cellular response or basal activation, which may impact overall protein and peptide identifications. In a previous study, we developed and validated an SP3 label-free peptide preparation method using the Biomek i5. The same lysate, prepared on different days using the automated sample handler, was found to be reproducible.^65^

**Figure 6:**
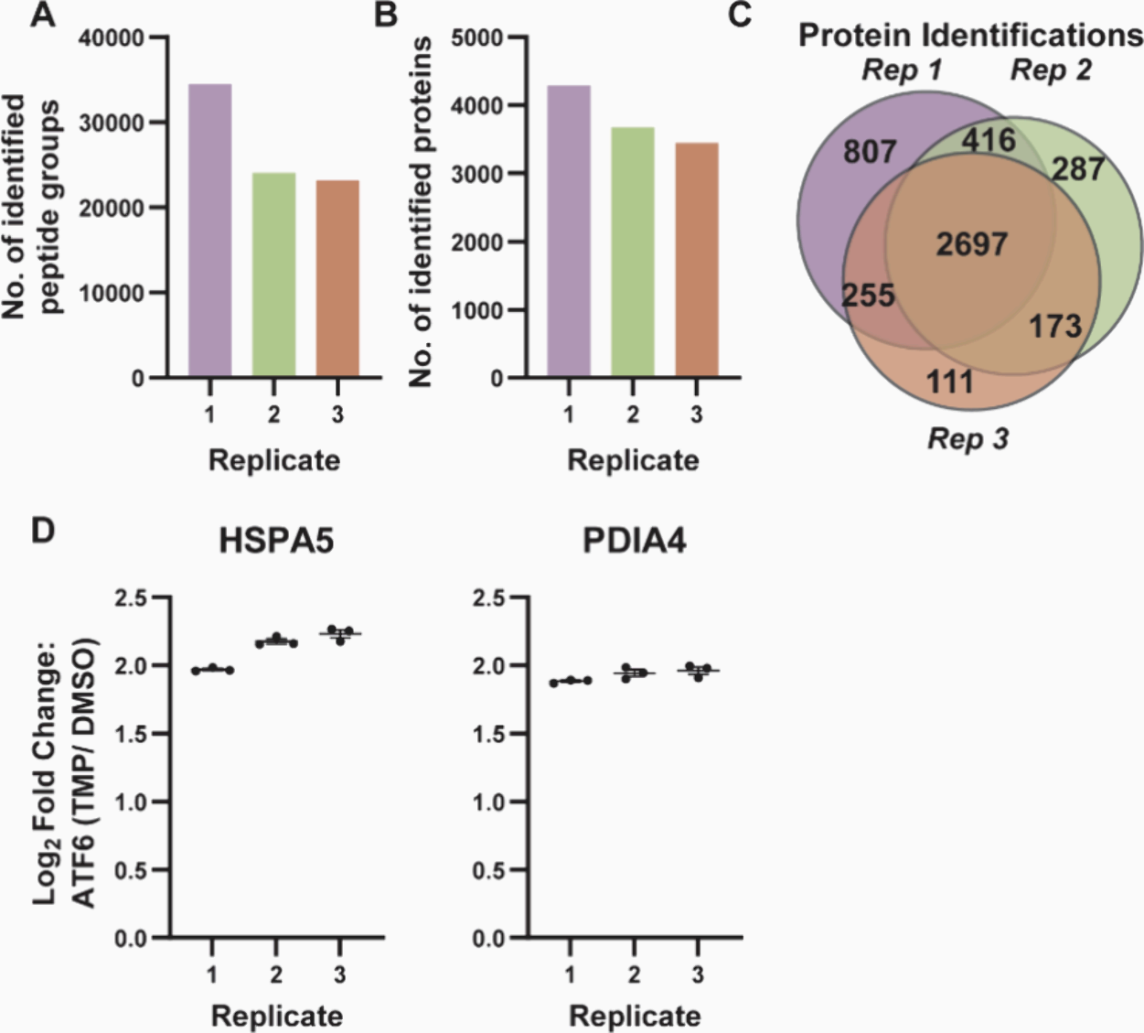
Validating the reproducibility of the developed Biomek i5 automated TMT labeling protocol. (A, B) Bar graphs showing the number of peptide groups and proteins detected across all TMT channels in the three separate replicates, each containing different biological replicates of activated cell lysate (*n* = 9). (C) Venn diagram depicting the unique and shared proteins identified across the three replicates. (D) Log_2_ fold change of HSPA5 and PDIA4, core ATF6 target proteins, across the three replicates. Error bars represent the mean ± SEM.

## Conclusions

Here we introduce an automated workflow designed to expedite the acquisition of TMT based proteomics data. Leveraging SP3 paramagnetic beads, our protocol efficiently converts normalized cell lysates into reduced, alkylated, and TMT-labeled peptides with minimal user intervention. While users must prepare reagents for loading onto the Biomek, the hands-on time is significantly reduced and remains consistent for up to 96 samples. The Biomek-assisted protocol requires approximately 2.8 h of manual intervention to generate 96 TMT-labeled samples, compared to 24 h for the fully manual protocol. The ability to process 96 samples in tandem on the Biomek provides considerable time savings over existing methods on other commercial platforms. Additionally, we demonstrate that the data quality achieved with our automated pipeline is improved by increasing the dynamic range of TMT quantification changes. This approach enhances data acquisition efficiency without necessitating extensive hands-on training or experience.

We believe our automated protocol will be amendable to other proteomic applications such as glycoproteomics^66^ and phosphoproteomics,^10^ due to SP3 bead compatibility with these PTMs. Furthermore, as additional multiplexing options become available, such as 32-plex and 35-plex,^23^ they can be integrated into this workflow. The 96-well format utilized in our method would allow for three 32-plex sets per plate compared to only two 35-plex sets. Additionally, previous studies have demonstrated the automation of sample preparation for tissue and serum/plasma samples using liquid handlers, suggesting that our platform would be amenable to these sample types as well.^14,17^

## Supplementary Material











## Notes

The following files are available free of charge. The Supporting Information is available free of charge at https://pubs.acs.org/doi/10.1021/acs.jproteome.5c00124.

Verification of UPR activation via Western blot (Figure S1); normalization of TMT protein abundances (Figure S2); identification of UPR markers for XBP 1s and PERK 18 h comparing biomek and manual (Figure S3) (PDF)
Estimation of hands-on time vs waiting periods for different sample preparation times (Table S1) (XLSX)
Proteome discoverer search file for manual sample preparation (Table S2) (XLSX)
Proteome discoverer search file for Biomek sample preparation (Table S3) (XLSX)
Example of Biomek deck layouts during 96 sample preparation (Supporting File 1) (PDF)

## Abbreviations

TMT: Tandem Mass Tags
SP3: single-pot solid-phase-enhanced sample preparation
UPR: unfolded protein response
MS: mass spectrometry
CV: coefficient of variation
LC: liquid chromatography
IAA: iodoacetamide
TCEP: tris­(2-carboxyethyl)­phosphine
TMP: trimethoprim
DOX: doxycycline
MudPIT: multidimensional protein identification technology
DDA: data dependent acquisition
PBS: phosphate buffered saline
EDTA: ethylenediaminetetraacetic acid
RIPA: radioimmunoprecipitation assay
ACN: acetonitrile
FA: formic acid
FDR: false discovery rate
FKBP: FK506-binding protein
dDHFR: destabilized DHFR
HEPA: high-efficiency particulate air

## References

[1] Shen Y., Timsina J., Heo G., Beric A., Ali M., Wang C., Yang C., Wang Y., Western D., Liu M., Gorijala P., Budde J., Do A., Liu H., Gordon B., Llibre-Guerra J. J., Joseph-Mathurin N., Perrin R. J., Maschi D., Wyss-Coray T., Pastor P., Renton A. E., Surace E. I., Johnson E. C. B., Levey A. I., Alvarez I., Levin J., Ringman J. M., Allegri R. F., Seyfried N., Day G. S., Wu Q., Fernández M. V., Tarawneh R., McDade E., Morris J. C., Bateman R. J., Goate A., Noble J. M., Day G. S., Graff-Radford N. R., Voglein J., Allegri R., Mendez P. C., Surace E., Berman S. B., Ikonomovic S., Nadkarni N., Lopera F., Ramirez L., Aguillon D., Leon Y., Ramos C., Alzate D., Baena A., Londono N., Mathias Jucker S. M., Laske C., Kuder-Buletta E., Graber-Sultan S., Preische O., Hofmann A., Ikeuchi T., Kasuga K., Niimi Y., Ishii K., Senda M., Sanchez-Valle R., Rosa-Neto P., Fox N., Cash D., Lee J. H., Roh J. H., Riddle M., Menard W., Bodge C., Surti M., Takada L. T., Farlow M., Chhatwal J. P., Sanchez-Gonzalez V. J., Orozco-Barajas M., Goate A., Renton A., Esposito B., Karch C. M., Marsh J., Cruchaga C., Fernandez V., Gordon B. A., Fagan A. M., Jerome G., Herries E., Llibre-Guerra J., Levey A. I., Johnson E. C. B., Seyfried N. T., Schofield P. R., Brooks W., Bechara J., Bateman R. J., McDade E., Hassenstab J., Perrin R. J., Franklin E., Benzinger T. L. S., Chen A., Chen C., Flores S., Friedrichsen N., Hantler N., Hornbeck R., Jarman S., Keefe S., Koudelis D., Massoumzadeh P., McCullough A., McKay N., Nicklaus J., Pulizos C., Wang Q., Mishall S., Sabaredzovic E., Deng E., Candela M., Smith H., Hobbs D., Scott J., Levin J., Xiong C., Wang P., Xu X., Li Y., Gremminger E., Ma Y., Bui R., Lu R., Martins R., Sosa Ortiz A. L., Daniels A., Courtney L., Mori H., Supnet-Bell C., Xu J., Ringman J., Ibanez L., Sung Y. J., Cruchaga C. (2024). CSF Proteomics Identifies
Early Changes in Autosomal Dominant Alzheimer’s Disease. Cell.

[2] Nusinow D. P., Szpyt J., Ghandi M., Rose C. M., McDonald E. R., Kalocsay M., Jané-Valbuena J., Gelfand E., Schweppe D. K., Jedrychowski M., Golji J., Porter D. A., Rejtar T., Wang Y. K., Kryukov G. V., Stegmeier F., Erickson B. K., Garraway L. A., Sellers W. R., Gygi S. P. (2020). Quantitative
Proteomics of the Cancer Cell Line Encyclopedia. Cell.

[3] Collins B. C., Hunter C. L., Liu Y., Schilling B., Rosenberger G., Bader S. L., Chan D. W., Gibson B. W., Gingras A.-C., Held J. M., Hirayama-Kurogi M., Hou G., Krisp C., Larsen B., Lin L., Liu S., Molloy M. P., Moritz R. L., Ohtsuki S., Schlapbach R., Selevsek N., Thomas S. N., Tzeng S.-C., Zhang H., Aebersold R. (2017). Multi-Laboratory Assessment of Reproducibility, Qualitative
and Quantitative Performance of SWATH-Mass Spectrometry. Nat. Commun..

[4] Guzman U. H., Martinez-Val A., Ye Z., Damoc E., Arrey T. N., Pashkova A., Renuse S., Denisov E., Petzoldt J., Peterson A. C., Harking F., Østergaard O., Rydbirk R., Aznar S., Stewart H., Xuan Y., Hermanson D., Horning S., Hock C., Makarov A., Zabrouskov V., Olsen J. V. (2024). Ultra-Fast Label-Free Quantification
and Comprehensive Proteome Coverage with Narrow-Window Data-Independent
Acquisition. Nat. Biotechnol..

[5] Keele G. R., Zhang J.-G., Szpyt J., Korstanje R., Gygi S. P., Churchill G. A., Schweppe D. K. (2023). Global and Tissue-Specific
Aging Effects on Murine Proteomes. Cell Rep.

[6] Locke T. M., Fields R., Gizinski H., Otto G. M., MacEwen M. J. S., Rusnac D.-V., He P., Shechner D. M., McGann C. D., Berg M. D., Villen J., Sancak Y., Schweppe D. K. (2024). High-Throughput
Identification of Calcium-Regulated Proteins across Diverse Proteomes. Cell Rep.

[7] Varnavides G., Madern M., Anrather D., Hartl N., Reiter W., Hartl M. (2022). In Search of a Universal Method:
A Comparative Survey of Bottom-Up
Proteomics Sample Preparation Methods. J. Proteome
Res..

[8] Müller T., Kalxdorf M., Longuespée R., Kazdal D. N., Stenzinger A., Krijgsveld J. (2020). Automated
Sample Preparation with SP 3 for Low-input
Clinical Proteomics. Mol. Syst. Biol..

[9] Reilly L., Lara E., Ramos D., Li Z., Pantazis C. B., Stadler J., Santiana M., Roberts J., Faghri F., Hao Y., Nalls M. A., Narayan P., Liu Y., Singleton A. B., Cookson M. R., Ward M. E., Qi Y. A. (2023). A Fully Automated
FAIMS-DIA Mass Spectrometry-Based Proteomic Pipeline. Cell Rep. Methods.

[10] Leutert M., Rodríguez-Mias R. A., Fukuda N. K., Villén J. (2019). R2-P2 Rapid-robotic
Phosphoproteomics Enables Multidimensional Cell Signaling Studies. Mol. Syst. Biol..

[11] Mun D.-G., Joshi N. S., Budhraja R., Sachdeva G. S., Kang T., Bhat F. A., Ding H., Madden B. J., Zhong J., Pandey A. (2023). Automated Sample Preparation
Workflow for Tandem Mass
Tag-Based Proteomics. J. Am. Soc. Mass Spectrom..

[12] Fu Q., Kowalski M. P., Mastali M., Parker S. J., Sobhani K., van den
Broek I., Hunter C. L., Van Eyk J. E. (2018). Highly Reproducible
Automated Proteomics Sample Preparation Workflow for Quantitative
Mass Spectrometry. J. Proteome Res..

[13] Oliver N. C., Choi M. J., Arul A. B., Whitaker M. D., Robinson R. A. S. (2024). Establishing
Quality Control Metrics for Large-Scale Plasma Proteomic Sample Preparation. ACS Meas. Sci. Au.

[14] King C. D., Kapp K. L., Arul A. B., Choi M. J., Robinson R. A. S. (2022). Advancements
in Automation for Plasma Proteomics Sample Preparation. Mol. Omics.

[15] Hendricks N. G., Bhosale S. D., Keoseyan A. J., Ortiz J., Stotland A., Seyedmohammad S., Nguyen C. D. L., Bui J. T., Moradian A., Mockus S. M., Van Eyk J. E. (2024). An Inflection Point in High-Throughput
Proteomics with Orbitrap Astral: Analysis of Biofluids, Cells, and
Tissues. J. Proteome Res..

[16] Liu X., Gygi S. P., Paulo J. A. (2021). A Semiautomated
Paramagnetic Bead-Based
Platform for Isobaric Tag Sample Preparation. J. Am. Soc. Mass Spectrom..

[17] Gaun A., Lewis Hardell K. N., Olsson N., O’Brien J. J., Gollapudi S., Smith M., McAlister G., Huguet R., Keyser R., Buffenstein R., McAllister F. E. (2021). Automated 16-Plex Plasma Proteomics
with Real-Time
Search and Ion Mobility Mass Spectrometry Enables Large-Scale Profiling
in Naked Mole-Rats and Mice. J. Proteome Res..

[18] Waas M., Pereckas M., Jones Lipinski R. A., Ashwood C., Gundry R. L. (2019). SP2: Rapid
and Automatable Contaminant Removal from Peptide Samples for Proteomic
Analyses. J. Proteome Res..

[19] Blume J. E., Manning W. C., Troiano G., Hornburg D., Figa M., Hesterberg L., Platt T. L., Zhao X., Cuaresma R. A., Everley P. A., Ko M., Liou H., Mahoney M., Ferdosi S., Elgierari E. M., Stolarczyk C., Tangeysh B., Xia H., Benz R., Siddiqui A., Carr S. A., Ma P., Langer R., Farias V., Farokhzad O. C. (2020). Rapid, Deep and Precise Profiling
of the Plasma Proteome
with Multi-Nanoparticle Protein Corona. Nat.
Commun..

[20] Fu Q., Murray C. I., Karpov O. A., Van Eyk J. E. (2021). Automated Proteomic
Sample Preparation: The Key Component for High Throughput and Quantitative
Mass Spectrometry Analysis. Mass Spectrom. Rev..

[21] Li J., Van Vranken J. G., Pontano Vaites L., Schweppe D. K., Huttlin E. L., Etienne C., Nandhikonda P., Viner R., Robitaille A. M., Thompson A. H., Kuhn K., Pike I., Bomgarden R. D., Rogers J. C., Gygi S. P., Paulo J. A. (2020). TMTpro Reagents:
A Set of Isobaric Labeling Mass Tags Enables Simultaneous Proteome-Wide
Measurements across 16 Samples. Nat. Methods.

[22] Li J., Cai Z., Bomgarden R. D., Pike I., Kuhn K., Rogers J. C., Roberts T. M., Gygi S. P., Paulo J. A. (2021). TMTpro-18plex: The
Expanded and Complete Set of TMTpro Reagents for Sample Multiplexing. J. Proteome Res..

[23] Zuniga N. R., Frost D. C., Kuhn K., Shin M., Whitehouse R. L., Wei T.-Y., He Y., Dawson S. L., Pike I., Bomgarden R. D., Gygi S. P., Paulo J. A. (2024). Achieving a 35-Plex
Tandem Mass Tag Reagent Set through Deuterium Incorporation. J. Proteome Res..

[24] Hughes C. S., Moggridge S., Müller T., Sorensen P. H., Morin G. B., Krijgsveld J. (2019). Single-Pot,
Solid-Phase-Enhanced Sample Preparation
for Proteomics Experiments. Nat. Protoc..

[25] Matsumura Y., Nakazaki T., Kitamori K., Kure E., Shinohara K., Tsuchido Y., Yukawa S., Noguchi T., Yamamoto M., Nagao M. (2023). Development and Evaluation of the Automated Multipurpose Molecular
Testing System PCRpack for High-Throughput SARS-CoV-2 Testing. Microbiol. Spectr..

[26] Lehmann R., Severitt J. C., Roddelkopf T., Junginger S., Thurow K. (2016). Biomek Cell Workstation: A Variable
System for Automated
Cell Cultivation. SLAS Technol..

[27] Conforti J. M., Ziegler A. M., Worth C. S., Nambiar A. M., Bailey J. T., Taube J. H., Gallagher E. S. (2024). Differences
in Protein Capture by
SP3 and SP4 Demonstrate Mechanistic Insights of Proteomics Cleanup
Techniques. J. Proteome Res..

[28] Mansuri M. S., Bathla S., Lam T. T., Nairn A. C., Williams K. R. (2024). Optimal
Conditions for Carrying out Trypsin Digestions on Complex Proteomes:
From Bulk Samples to Single Cells. J. Proteomics.

[29] Zecha J., Satpathy S., Kanashova T., Avanessian S. C., Kane M. H., Clauser K. R., Mertins P., Carr S. A., Kuster B. (2019). TMT Labeling for the Masses: A Robust
and Cost-Efficient,
In-Solution Labeling Approach*­[S]. Mol. Cell.
Proteomics.

[30] Harding H. P., Zeng H., Zhang Y., Jungries R., Chung P., Plesken H., Sabatini D. D., Ron D. (2001). Diabetes Mellitus
and
Exocrine Pancreatic Dysfunction in Perk–/– Mice Reveals
a Role for Translational Control in Secretory Cell Survival. Mol. Cell.

[31] Kawasaki N., Asada R., Saito A., Kanemoto S., Imaizumi K. (2012). Obesity-Induced
Endoplasmic Reticulum Stress Causes Chronic Inflammation in Adipose
Tissue. Sci. Rep..

[32] Cabral-Miranda F., Tamburini G., Martinez G., Ardiles A. O., Medinas D. B., Gerakis Y., Hung M. D., Vidal R., Fuentealba M., Miedema T., Duran-Aniotz C., Diaz J., Ibaceta-Gonzalez C., Sabusap C. M., Bermedo-Garcia F., Mujica P., Adamson S., Vitangcol K., Huerta H., Zhang X., Nakamura T., Sardi S. P., Lipton S. A., Kennedy B. K., Henriquez J. P., Cárdenas J. C., Plate L., Palacios A. G., Hetz C. (2022). Unfolded Protein
Response IRE1/XBP1 Signaling Is Required for Healthy Mammalian Brain
Aging. EMBO J..

[33] Genovese I., Giamogante F., Barazzuol L., Battista T., Fiorillo A., Vicario M., D’Alessandro G., Cipriani R., Limatola C., Rossi D., Sorrentino V., Poser E., Mosca L., Squitieri F., Perluigi M., Arena A., van Petegem F., Tito C., Fazi F., Giorgi C., Calì T., Ilari A., Colotti G. (2020). Sorcin Is an Early Marker of Neurodegeneration,
Ca2+ Dysregulation and Endoplasmic Reticulum Stress Associated to
Neurodegenerative Diseases. Cell Death Dis..

[34] Bugallo R., Marlin E., Baltanás A., Toledo E., Ferrero R., Vinueza-Gavilanes R., Larrea L., Arrasate M., Aragón T. (2020). Fine Tuning
of the Unfolded Protein Response by ISRIB Improves Neuronal Survival
in a Model of Amyotrophic Lateral Sclerosis. Cell Death Dis..

[35] Bobrovnikova-Marjon E., Grigoriadou C., Pytel D., Zhang F., Ye J., Koumenis C., Cavener D., Diehl J. A. (2010). PERK Promotes Cancer
Cell Proliferation and Tumor Growth by Limiting Oxidative DNA Damage. Oncogene.

[36] Reich S., Nguyen C. D. L., Has C., Steltgens S., Soni H., Coman C., Freyberg M., Bichler A., Seifert N., Conrad D., Knobbe-Thomsen C. B., Tews B., Toedt G., Ahrends R., Medenbach J. (2020). A Multi-Omics
Analysis Reveals the Unfolded Protein Response Regulon and Stress-Induced
Resistance to Folate-Based Antimetabolites. Nat. Commun..

[37] Li C., Fan Q., Quan H., Nie M., Luo Y., Wang L. (2018). The Three
Branches of the Unfolded Protein Response Exhibit Differential Significance
in Breast Cancer Growth and Stemness. Exp. Cell
Res..

[38] Roy B. (1999). The Mammalian
Endoplasmic Reticulum Stress Response Element Consists of an Evolutionarily
Conserved Tripartite Structure and Interacts with a Novel Stress-Inducible
Complex. Nucleic Acids Res..

[39] Haze K., Yoshida H., Yanagi H., Yura T., Mori K. (1999). Mammalian
Transcription Factor ATF6 Is Synthesized as a Transmembrane Protein
and Activated by Proteolysis in Response to Endoplasmic Reticulum
Stress. Mol. Biol. Cell.

[40] Harding H. P., Zhang Y., Ron D. (1999). Protein Translation
and Folding Are
Coupled by an Endoplasmic-Reticulum- Resident Kinase. Nature.

[41] Cox J. S., Shamu C. E., Walter P. (1993). Transcriptional
Induction of Genes
Encoding Endoplasmic Reticulum Resident Proteins Requires a Transmembrane
Protein Kinase. Cell.

[42] Shoulders M. D., Ryno L. M., Genereux J. C., Moresco J. J., Tu P. G., Wu C., Yates J. R., Su A. I., Kelly J. W., Wiseman R. L. (2013). Stress-Independent
Activation of XBP1s and/or ATF6 Reveals Three Functionally Diverse
ER Proteostasis Environments. Cell Rep..

[43] Lee A.-H., Iwakoshi N. N., Glimcher L. H. (2003). XBP-1 Regulates a Subset of Endoplasmic
Reticulum Resident Chaperone Genes in the Unfolded Protein Response. Mol. Cell. Biol..

[44] Yamamoto K. (2004). Differential
Contributions of ATF6 and XBP1 to the Activation of Endoplasmic Reticulum
Stress-Responsive Cis-Acting Elements ERSE. UPRE and ERSE-II. J. Biochem. (Tokyo).

[45] Grandjean J. M. D., Plate L., Morimoto R. I., Bollong M. J., Powers E. T., Wiseman R. L. (2019). Deconvoluting Stress-Responsive
Proteostasis Signaling
Pathways for Pharmacologic Activation Using Targeted RNA Sequencing. ACS Chem. Biol..

[46] Davies J. P., Sivadas A., Keller K. R., Roman B. K., Wojcikiewicz R. J. H., Plate L. (2024). Expression of SARS-CoV-2
Nonstructural Proteins 3 and
4 Can Tune the Unfolded Protein Response in Cell Culture. J. Proteome Res..

[47] Wright M. T., Kouba L., Plate L. (2021). Thyroglobulin
Interactome Profiling
Defines Altered Proteostasis Topology Associated With Thyroid Dyshormonogenesis. Mol. Cell. Proteomics.

[48] Kim M., McDonald E. F., Sabusap C. M. P., Timalsina B., Joshi D., Hong J. S., Rab A., Sorscher E. J., Plate L. (2023). Elexacaftor/VX-445–Mediated CFTR Interactome Remodeling Reveals
Differential Correction Driven by Mutation-Specific Translational
Dynamics. J. Biol. Chem..

[49] Almasy K. M., Davies J. P., Plate L. (2021). Comparative
Host Interactomes of
the SARS-CoV-2 Nonstructural Protein 3 and Human Coronavirus Homologs. Mol. Cell. Proteomics.

[50] Kind D., Baskaran P., Ramirez F., Giner M., Hayes M., Santacruz D., Koss C. K., el Kasmi K. C., Wijayawardena B., Viollet C. (2022). Automation Enables High-Throughput
and Reproducible
Single-Cell Transcriptomics Library Preparation. SLAS Technol..

[51] Ryan M. J., Grant-St James A., Lawler N. G., Fear M. W., Raby E., Wood F. M., Maker G. L., Wist J., Holmes E., Nicholson J. K., Whiley L., Gray N. (2023). Comprehensive
Lipidomic
Workflow for Multicohort Population Phenotyping Using Stable Isotope
Dilution Targeted Liquid Chromatography-Mass Spectrometry. J. Proteome Res..

[52] Bowser B. L., Patterson K. L., Robinson R. A. (2023). Evaluating cPILOT Data toward Quality
Control Implementation. J. Am. Soc. Mass Spectrom..

[53] Smith P. K., Krohn R. I., Hermanson G. T., Mallia A. K., Gartner F. H., Provenzano M. D., Fujimoto E. K., Goeke N. M., Olson B. J., Klenk D. C. (1985). Measurement of Protein Using Bicinchoninic Acid. Anal. Biochem..

[54] Sielaff M., Kuharev J., Bohn T., Hahlbrock J., Bopp T., Tenzer S., Distler U. (2017). Evaluation
of FASP,
SP3, and iST Protocols for Proteomic Sample Preparation in the Low
Microgram Range. J. Proteome Res..

[55] Lu P. D., Jousse C., Marciniak S. J., Zhang Y., Novoa I., Scheuner D., Kaufman R. J., Ron D., Harding H. P. (2004). Cytoprotection
by Pre-Emptive Conditional Phosphorylation of Translation Initiation
Factor 2. EMBO J..

[56] Webb K. J., Xu T., Park S. K., Yates J. R. (2013). Modified MuDPIT Separation Identified
4488 Proteins in a System-Wide Analysis of Quiescence in Yeast. J. Proteome Res..

[57] Fournier M. L., Gilmore J. M., Martin-Brown S. A., Washburn M. P. (2007). Multidimensional
Separations-Based Shotgun Proteomics. Chem.
Rev..

[58] Washburn M. P., Ulaszek R., Deciu C., Schieltz D. M., Yates J. R. (2002). Analysis
of Quantitative Proteomic Data Generated via Multidimensional Protein
Identification Technology. Anal. Chem..

[59] Wolters D.
A., Washburn M. P., Yates J. R. (2001). An Automated Multidimensional Protein
Identification Technology for Shotgun Proteomics. Anal. Chem..

[60] Frankenfield A. M., Ni J., Ahmed M., Hao L. (2022). Protein Contaminants Matter: Building
Universal Protein Contaminant Libraries for DDA and DIA Proteomics. J. Proteome Res..

[61] Brenes A. J. (2024). Calculating
and Reporting Coefficients of Variation for DIA-Based Proteomics. J. Proteome Res..

[62] Vandermarliere E., Mueller M., Martens L. (2013). Getting Intimate
with Trypsin, the
Leading Protease in Proteomics. Mass Spectrom.
Rev..

[63] Cantrell L. S., Gletten R. B., Schey K. L. (2023). Proteome Remodeling of the Eye Lens
at 50 Years Identified With Data-Independent Acquisition. Mol. Cell. Proteomics.

[64] Hughes C. S., Foehr S., Garfield D. A., Furlong E. E., Steinmetz L. M., Krijgsveld J. (2014). Ultrasensitive
Proteome Analysis Using Paramagnetic
Bead Technology. Mol. Syst. Biol..

[65] Barny L. A., Hermanson J. N., Garcia S. K., Stauffer P. E., Plate L. (2025). Dissecting
Branch-Specific Unfolded Protein Response Activation in Drug-Tolerant
BRAF-Mutant Melanoma Using Data-Independent Acquisition Mass Spectrometry. bioRxiv.

[66] Bagdonaite I., Malaker S. A., Polasky D. A., Riley N. M., Schjoldager K., Vakhrushev S. Y., Halim A., Aoki-Kinoshita K. F., Nesvizhskii A. I., Bertozzi C. R., Wandall H. H., Parker B. L., Thaysen-Andersen M., Scott N. E. (2022). Glycoproteomics. Nat. Rev. Methods
Primer.

