# Supplementary material for: Development of an Adaptive, Economical, and Easy-to-Use SP3-TMT Automated Sample Preparation Workflow for Quantitative Proteomics
Source: J Proteome Res. 2025 May 27;24(6):2996–3006. doi: 10.1021/acs.jproteome.5c00124 (PMC12150330; doi:10.1021/acs.jproteome.5c00124)
Supplement: Supplementary file 1 [file pr5c00124_si_001.pdf]

Supporting Information for:

Development of an Adaptive, Economical, and Easy-to-Use SP3-TMT Automated Sample Preparation Workflow for Quantitative Proteomics

Jake N. Hermanson[1‡], Lea A. Barny[2‡], Lars Plate[1,2,3,4*]

[1] Department of Biological Sciences, Vanderbilt University Nashville, Tennessee

[2] Program in Chemical and Physical Biology, Vanderbilt University Nashville, Tennessee

[3] Department of Chemistry, Vanderbilt University Nashville, Tennessee

[4] Department of Pathology, Microbiology and Immunology, Vanderbilt University Medical Center, Nashville, Tennessee

[*]Corresponding author: lars.plate@vanderbilt.edu

[‡] These Authors Contributed Equally

Supporting Figures

Figure S1 Western blot validation of activated HEK^{293}DAX and Fv2e-PERK cells.

Figure S2 Normalization of TMT protein abundances.

Figure S3 XBP1s/ PERK target protein quantification, coefficient percentages and correlation plot analysis.

Supporting Tables (supplied as separate Excel files)

Table S1 Comparison of automated (Accelerome/ Biomek i5) and manual protocol completion times.

Table S2 Proteins identified via the manual preparation.

Table S3 Proteins identified via the Biomek i5 preparation.

Supporting Files (supplied as separate PDF file)

File S1 Deck setups for Biomek sample preparation (PDF).

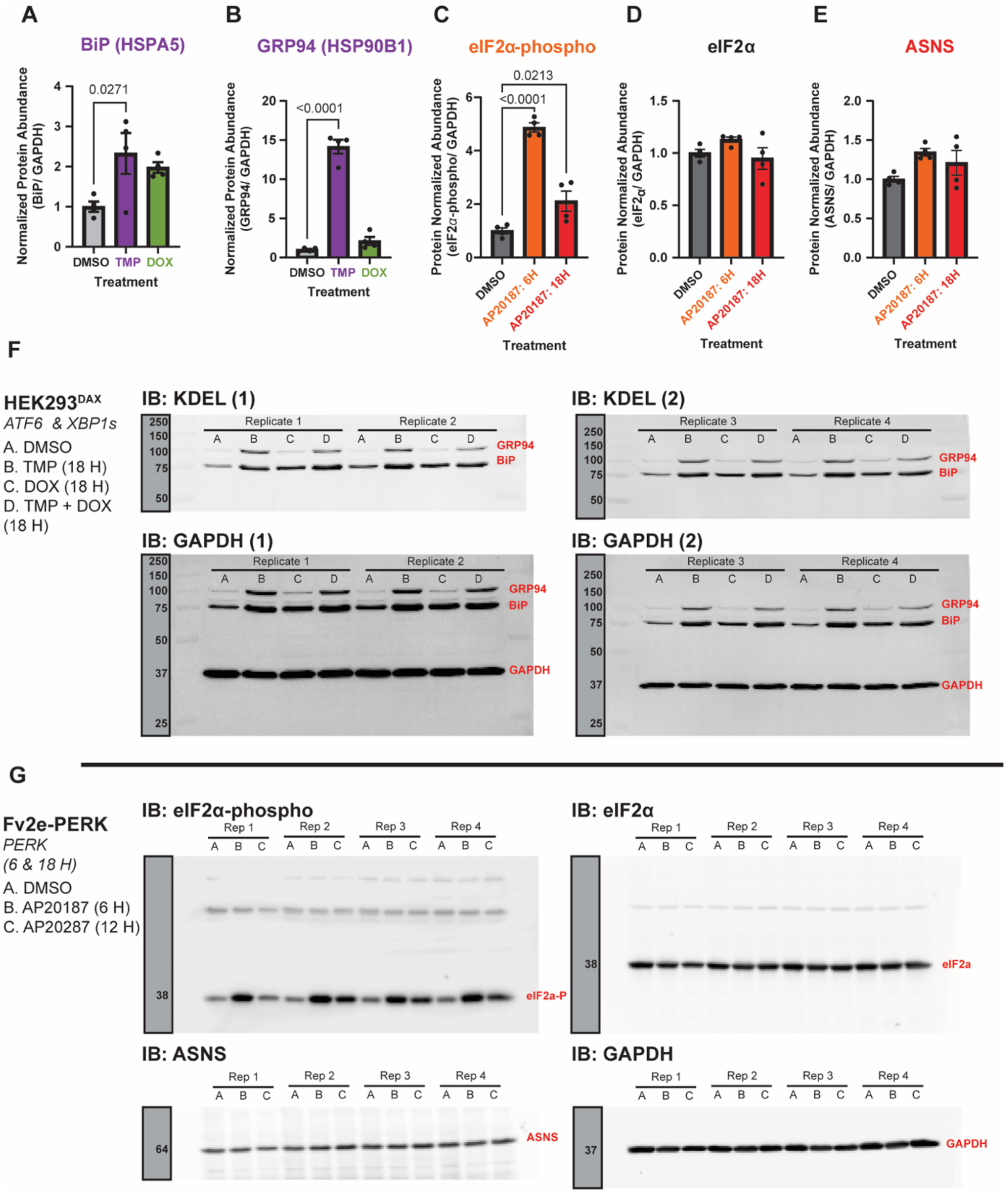

Figure S1. Western blot validation of activated HEK^{293}DAX and Fv2e-PERK cell activation. To upregulate the transcription factors, ATF6 and XBP1s, HEK^{293}DAX cells were treated with TMP (10 µM) and DOX (1 µg/mL), respectively for 16 hours. **(A/B)** BiP and HSP90B1 target proteins were utilized to validate activation of ATF6. **(C/D)** Fv2e-PERK cells were alternatively treated with AP20187 (5 nM) for 6 and 18 hours resulting in the phosphorylation of eIF2α (visualized via western blot). **(E)** ASNS was additionally utilized to probe for PERK activation. **(F)** Western blots for HEK^{293} DAX cell line activation of ATF6 and XBP1s. **(G)** Western blots of Fv2e-PERK cell activation for 6 and 18 hours.

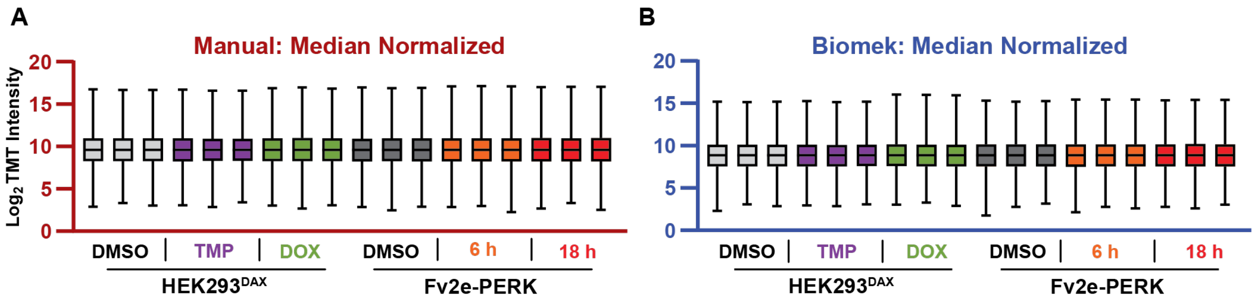

Figure S2. Normalization of TMT protein abundances. (A/B) Distribution of protein abundances post median normalization for the manual **(A)** and Biomek **(B)** sample preparation protocol.

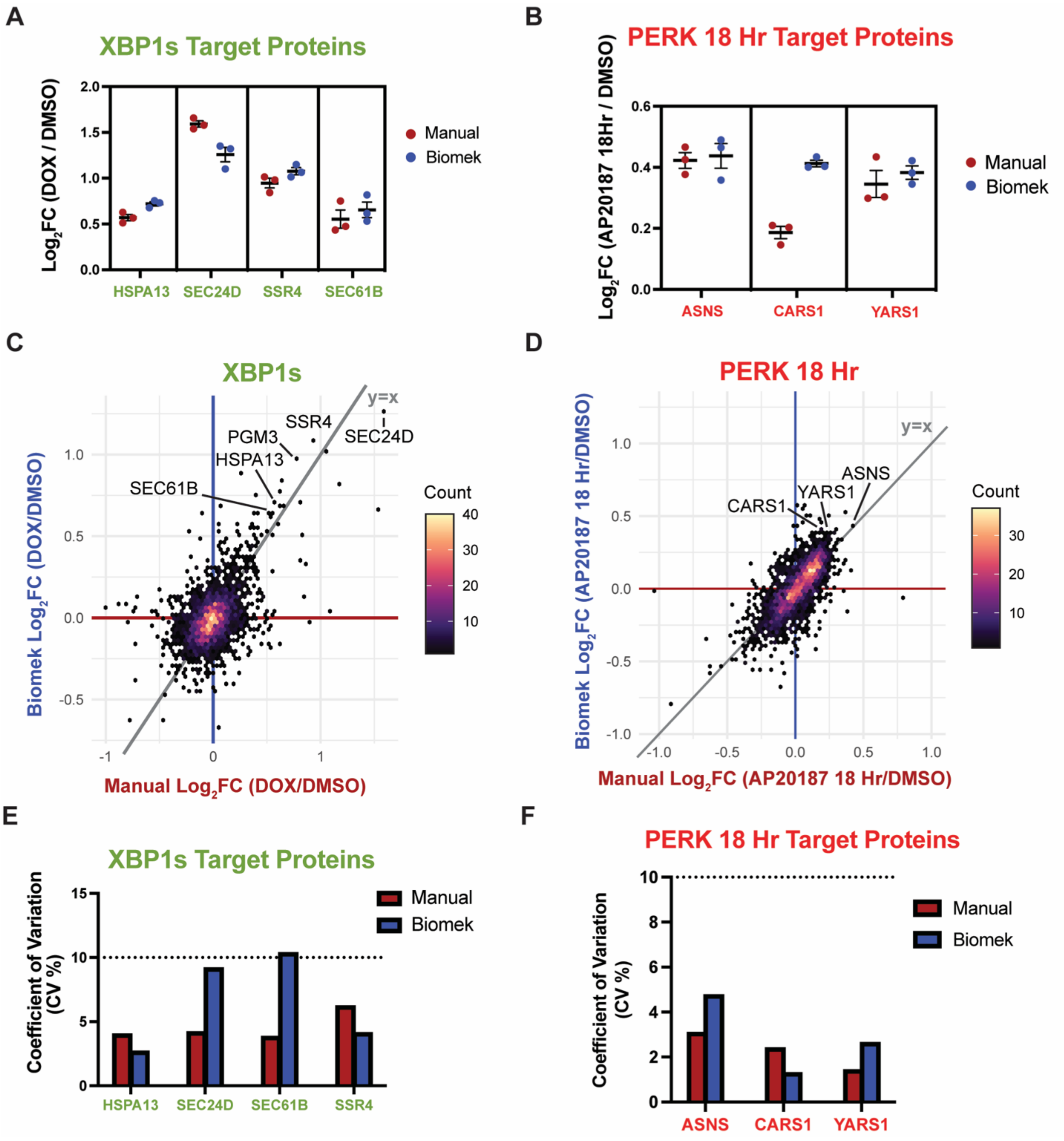

Figure S3. Identification of core UPR markers for both XBP1s and PERK 18 Hr in the Biomek and manual sample preparation. Log_2 fold change of core **(A)** XBP1s and **(B)** PERK 18 Hr between the two methods. Correlation plot of Log_2 fold change of **(C)** XBP1s and **(D)** PERK 18 Hr comparing the two sample preparations. Coefficient of variation of select **(E)** XBP1s target and select **(F)** PERK targets.
