# Supplementary material for: Development of an Adaptive, Economical, and Easy-to-Use SP3-TMT Automated Sample Preparation Workflow for Quantitative Proteomics
Source: J Proteome Res. 2025 May 27;24(6):2996–3006. doi: 10.1021/acs.jproteome.5c00124 (PMC12150330; doi:10.1021/acs.jproteome.5c00124)
Supplement: Supplementary file 5 [file pr5c00124_si_005.pdf]

Labware Setup

This report shows where to place labware on the deck. The quantities and types of labware are listed below.
Start Time: (Print Time: 1/13/2025 7:50:19 PM)

Method: GFP Pull Down_V1.1_LowVol2_21Jan2024, **Project:** Biomek i5 MC

Overall Setup Information

The left pod should have no tips loaded.

Tips and Empty Labware Deck Setup

Ensure that labware is placed on the deck in the positions below.

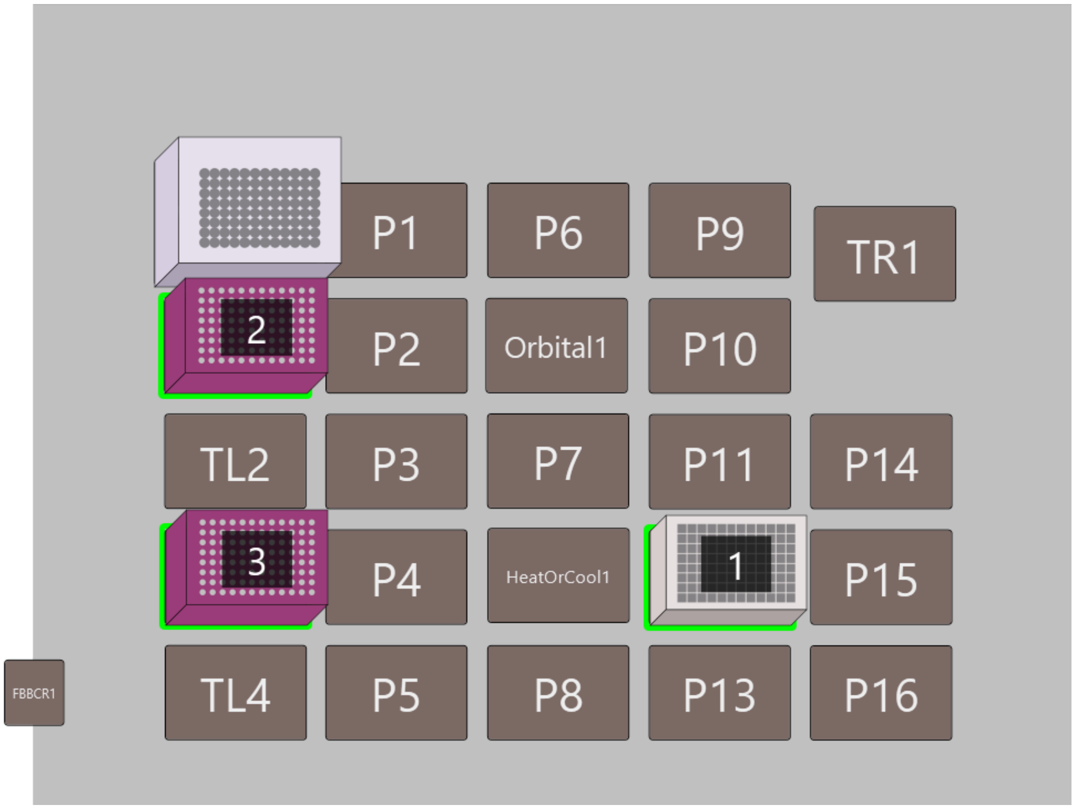

#	Name	Type	Position	Stack Depth
1	Dest	ab_0932	P12	1
2	Water_Tips	BC90	TL1	1
3	Sample_Tips	BC90	TL3	1

2. Samples and Reagents

Labware Setup for Sample (Bio_RadPCR96)

	1	2	3	4	5	6	7	8	9	10	11	12
A	Sample 45 uL	Sample 45 uL	Sample 45 uL	Sample 45 uL	Sample 45 uL	Sample 45 uL	Sample 45 uL	Sample 45 uL	Sample 45 uL	Sample 45 uL	Sample 45 uL	Sample 45 uL
B	Sample 45 uL	Sample 45 uL	Sample 45 uL	Sample 45 uL	Sample 45 uL	Sample 45 uL	Sample 45 uL	Sample 45 uL	Sample 45 uL	Sample 45 uL	Sample 45 uL	Sample 45 uL
C	Sample 45 uL	Sample 45 uL	Sample 45 uL	Sample 45 uL	Sample 45 uL	Sample 45 uL	Sample 45 uL	Sample 45 uL	Sample 45 uL	Sample 45 uL	Sample 45 uL	Sample 45 uL
D	Sample 45 uL	Sample 45 uL	Sample 45 uL	Sample 45 uL	Sample 45 uL	Sample 45 uL	Sample 45 uL	Sample 45 uL	Sample 45 uL	Sample 45 uL	Sample 45 uL	Sample 45 uL
E	Sample 45 uL	Sample 45 uL	Sample 45 uL	Sample 45 uL	Sample 45 uL	Sample 45 uL	Sample 45 uL	Sample 45 uL	Sample 45 uL	Sample 45 uL	Sample 45 uL	Sample 45 uL
F	Sample 45 uL	Sample 45 uL	Sample 45 uL	Sample 45 uL	Sample 45 uL	Sample 45 uL	Sample 45 uL	Sample 45 uL	Sample 45 uL	Sample 45 uL	Sample 45 uL	Sample 45 uL
G	Sample 45 uL	Sample 45 uL	Sample 45 uL	Sample 45 uL	Sample 45 uL	Sample 45 uL	Sample 45 uL	Sample 45 uL	Sample 45 uL	Sample 45 uL	Sample 45 uL	Sample 45 uL
H	Sample 45 uL	Sample 45 uL	Sample 45 uL	Sample 45 uL	Sample 45 uL	Sample 45 uL	Sample 45 uL	Sample 45 uL	Sample 45 uL	Sample 45 uL	Sample 45 uL	Sample 45 uL

Place Sample(Bio_RadPCR96) on position P13

Samples and Reagents Deck Setup

Ensure that labware is placed on the deck in the positions below.

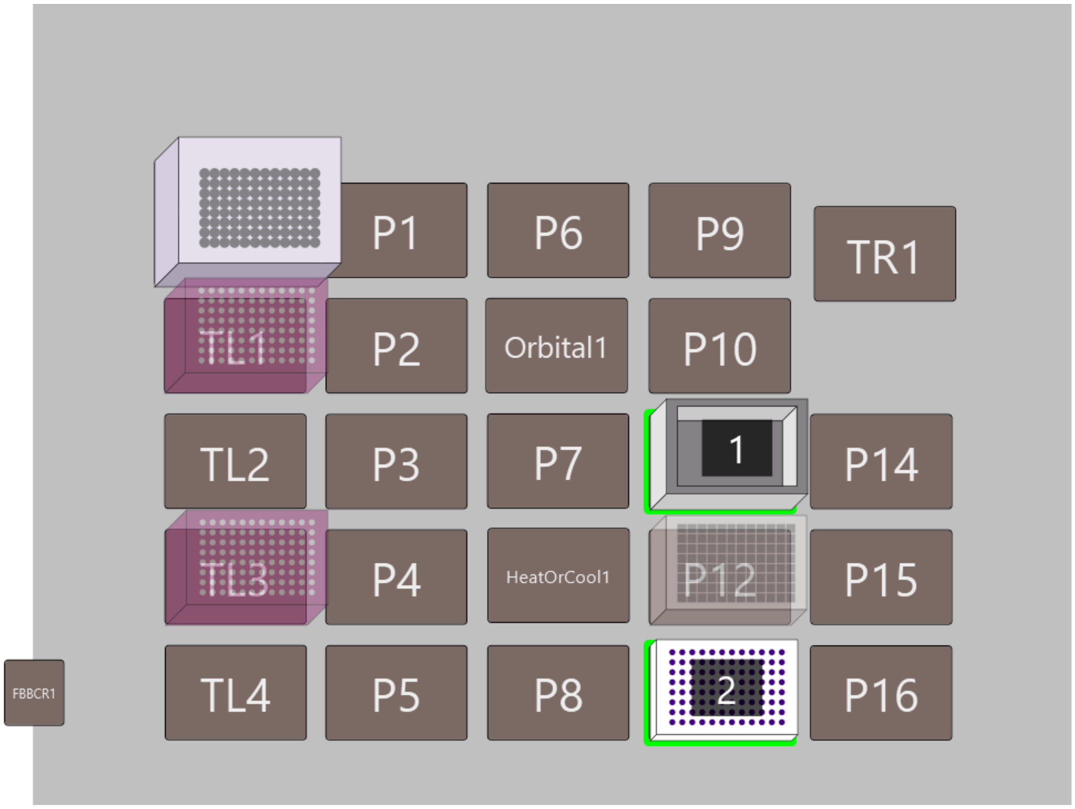

#	Name	Type	Position	Stack Depth
1	Water	AgilentReservoir	P11	1
2	Sample	Bio_RadPCR96	P13	1

Final Deck Setup

Below is the final deck layout. Ensure that all the highlighted labware are in the specified positions.

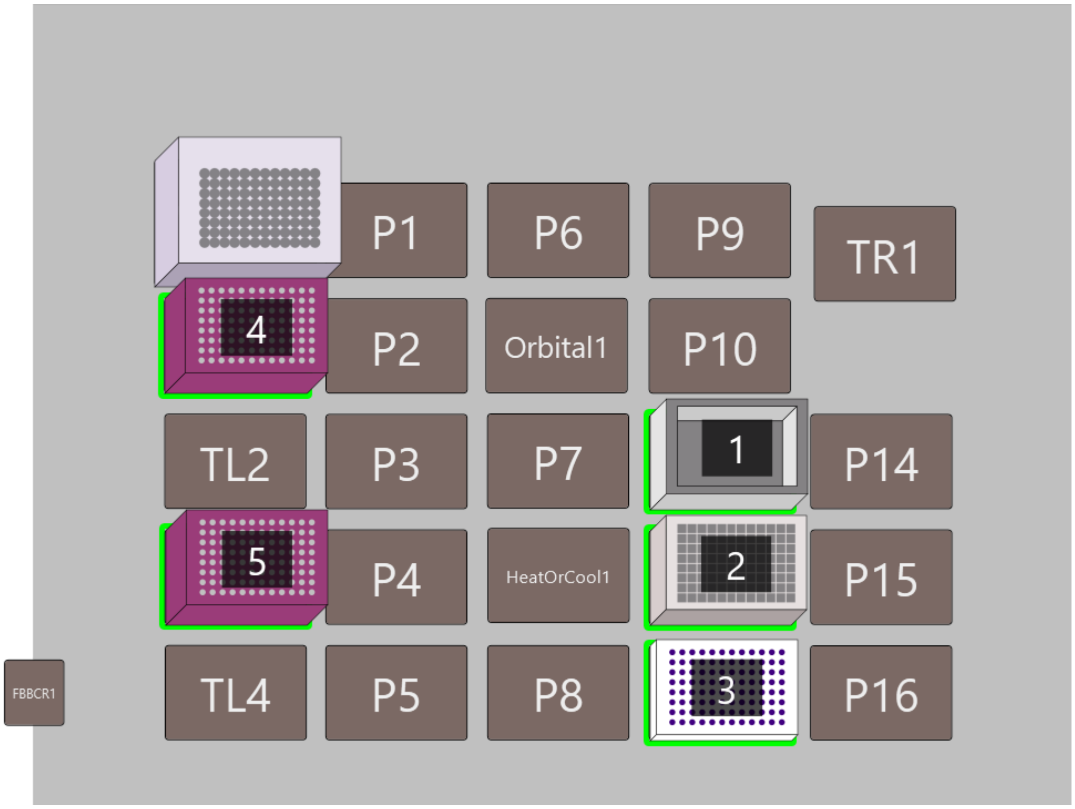

#	Name	Type	Position	Stack Depth
1	Water	AgilentReservoir	P11	1
2	Dest	ab_0932	P12	1
3	Sample	Bio_RadPCR96	P13	1
4	Water_Tips	BC90	TL1	1
5	Sample_Tips	BC90	TL3	1

Method Details

Labware Setup

This report shows where to place labware on the deck. The quantities and types of labware are listed below.
Start Time: (Print Time: 1/14/2025 10:21:40 AM)

Method: SP3 Bead_V1.1_Pauses_50ul, **Project:** Biomek i5 MC

Overall Setup Information

The left pod should have no tips loaded.

1. New Labware Group

Labware Setup for Sample (ab_0932)

	1	2	3	4	5	6	7	8	9	10	11	12
A	20 ug protein 70 uL	20 ug protein 70 uL	20 ug protein 70 uL	20 ug protein 70 uL	20 ug protein 70 uL	20 ug protein 70 uL	20 ug protein 70 uL	20 ug protein 70 uL	20 ug protein 70 uL	20 ug protein 70 uL	20 ug protein 70 uL	20 ug protein 70 uL
B	20 ug protein 70 uL	20 ug protein 70 uL	20 ug protein 70 uL	20 ug protein 70 uL	20 ug protein 70 uL	20 ug protein 70 uL	20 ug protein 70 uL	20 ug protein 70 uL	20 ug protein 70 uL	20 ug protein 70 uL	20 ug protein 70 uL	20 ug protein 70 uL
C	20 ug protein 70 uL	20 ug protein 70 uL	20 ug protein 70 uL	20 ug protein 70 uL	20 ug protein 70 uL	20 ug protein 70 uL	20 ug protein 70 uL	20 ug protein 70 uL	20 ug protein 70 uL	20 ug protein 70 uL	20 ug protein 70 uL	20 ug protein 70 uL
D	20 ug protein 70 uL	20 ug protein 70 uL	20 ug protein 70 uL	20 ug protein 70 uL	20 ug protein 70 uL	20 ug protein 70 uL	20 ug protein 70 uL	20 ug protein 70 uL	20 ug protein 70 uL	20 ug protein 70 uL	20 ug protein 70 uL	20 ug protein 70 uL
E	20 ug protein 70 uL	20 ug protein 70 uL	20 ug protein 70 uL	20 ug protein 70 uL	20 ug protein 70 uL	20 ug protein 70 uL	20 ug protein 70 uL	20 ug protein 70 uL	20 ug protein 70 uL	20 ug protein 70 uL	20 ug protein 70 uL	20 ug protein 70 uL
F	20 ug protein 70 uL	20 ug protein 70 uL	20 ug protein 70 uL	20 ug protein 70 uL	20 ug protein 70 uL	20 ug protein 70 uL	20 ug protein 70 uL	20 ug protein 70 uL	20 ug protein 70 uL	20 ug protein 70 uL	20 ug protein 70 uL	20 ug protein 70 uL
G	20 ug protein 70 uL	20 ug protein 70 uL	20 ug protein 70 uL	20 ug protein 70 uL	20 ug protein 70 uL	20 ug protein 70 uL	20 ug protein 70 uL	20 ug protein 70 uL	20 ug protein 70 uL	20 ug protein 70 uL	20 ug protein 70 uL	20 ug protein 70 uL
H	20 ug protein 70 uL	20 ug protein 70 uL	20 ug protein 70 uL	20 ug protein 70 uL	20 ug protein 70 uL	20 ug protein 70 uL	20 ug protein 70 uL	20 ug protein 70 uL	20 ug protein 70 uL	20 ug protein 70 uL	20 ug protein 70 uL	20 ug protein 70 uL

Place Sample(ab_0932) on position P11

Labware Setup for Reagents (ab_0932)

	1	2	3	4	5	6	7	8	9	10	11	12
A	100 mM DTT 113.8uL	200 mM IAA 133uL										
B	100 mM DTT 113.8uL	200 mM IAA 133uL										
C	100 mM DTT 113.8uL	200 mM IAA 133uL										
D	100 mM DTT 113.8uL	200 mM IAA 133uL										
E	100 mM DTT 113.8uL	200 mM IAA 133uL										
F	100 mM DTT 113.8uL	200 mM IAA 133uL										
G	100 mM DTT 113.8uL	200 mM IAA 133uL										
H	100 mM DTT 113.8uL	200 mM IAA 133uL										

Place Reagents(ab_0932) on position P12

New Labware Group Deck Setup

Ensure that labware is placed on the deck in the positions below.

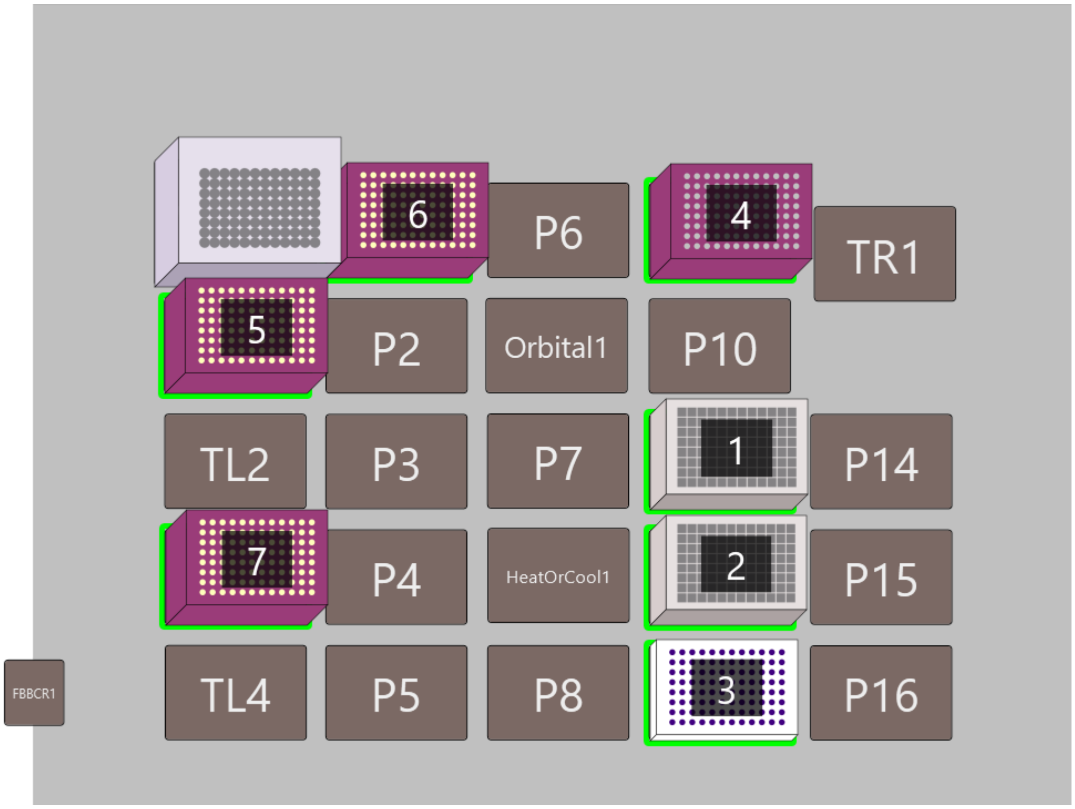

#	Name	Type	Position	Stack Depth
1	Sample	ab_0932	P11	1
2	Reagents	ab_0932	P12	1
3	Gel	Bio_RadPCR96	P13	1
4	Gel_Tips	BC90	P9	1
5	DTT_Tips1	BC50F	TL1	1
6	DTT_Tips2	BC50F	P1	1
7	IAA_Tips	BC50F	TL3	1

Final Deck Setup

Below is the final deck layout. Ensure that all the highlighted labware are in the specified positions.

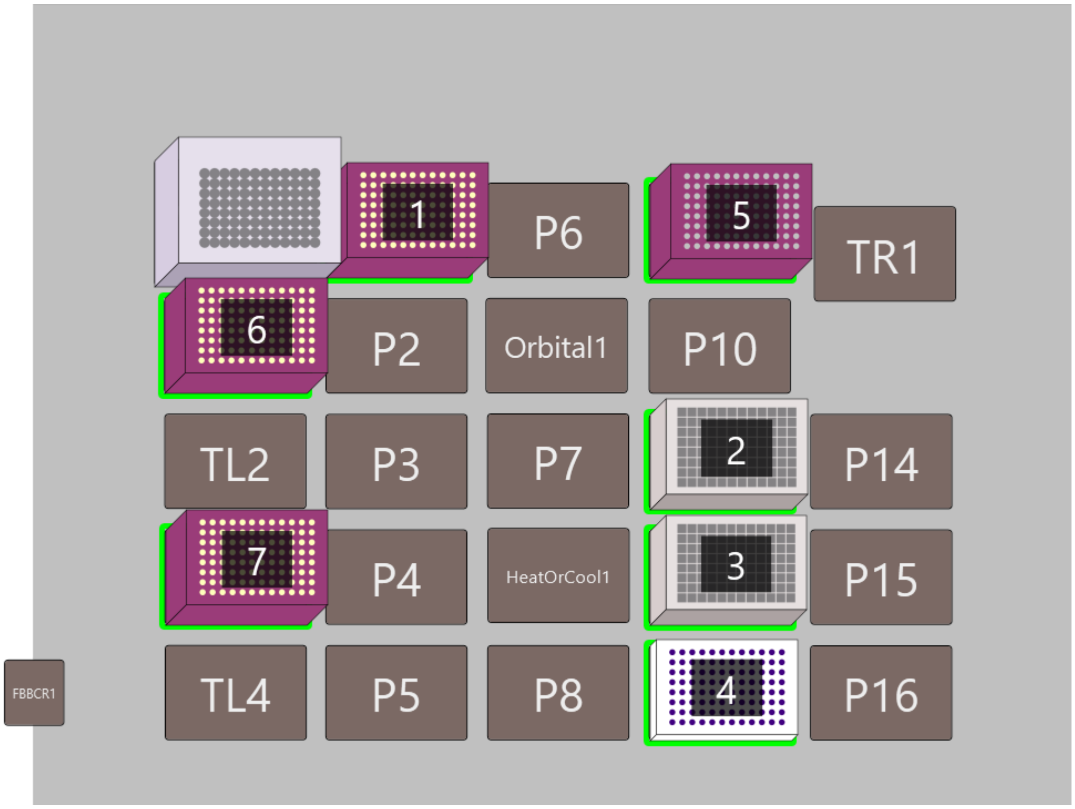

Labware Setup

This report shows where to place labware on the deck. The quantities and types of labware are listed below.
Start Time: (Print Time: 1/14/2025 10:52:50 AM)

Method: SP3 Bead_V1.1_Pauses_50ul, **Project:** Biomek i5 MC

Overall Setup Information

The left pod should have no tips loaded.

1. New Labware Group

Labware Setup for EtOH (AgilentReservoir)

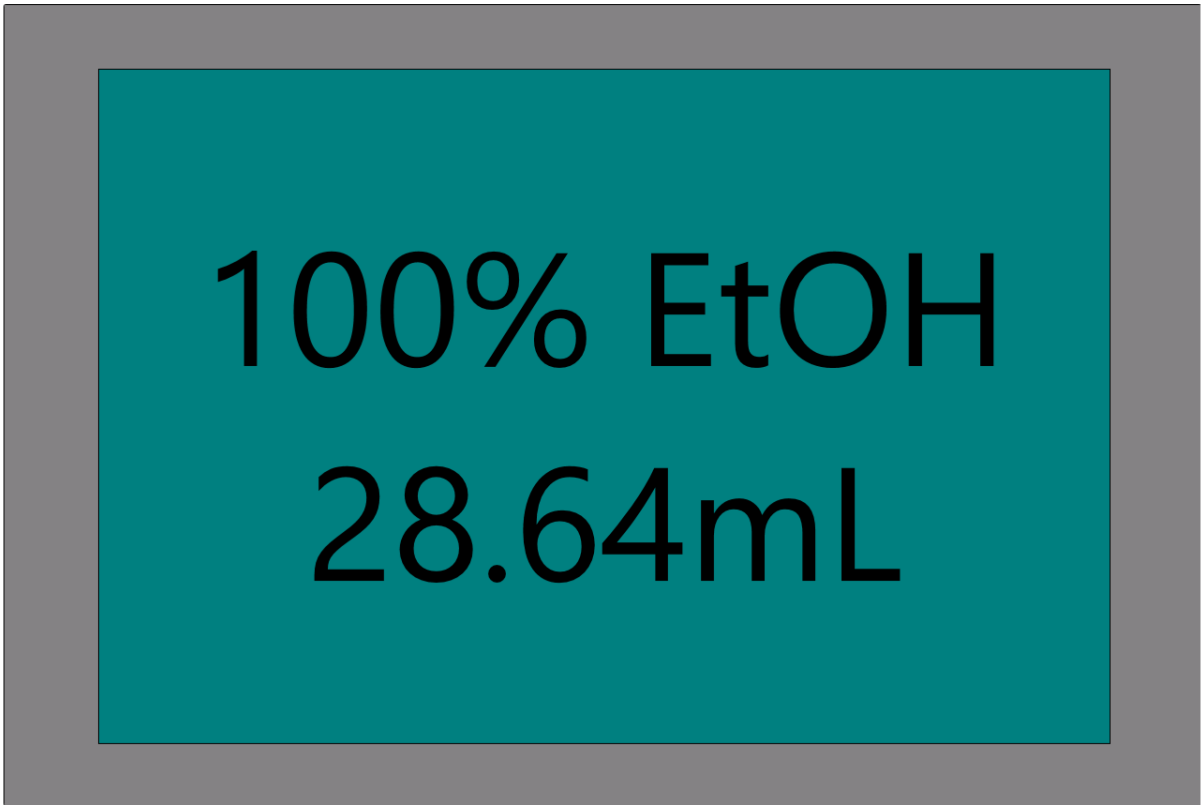

Place EtOH(AgilentReservoir) on position P8

Labware Setup for 80% EtOH (AgilentReservoir)

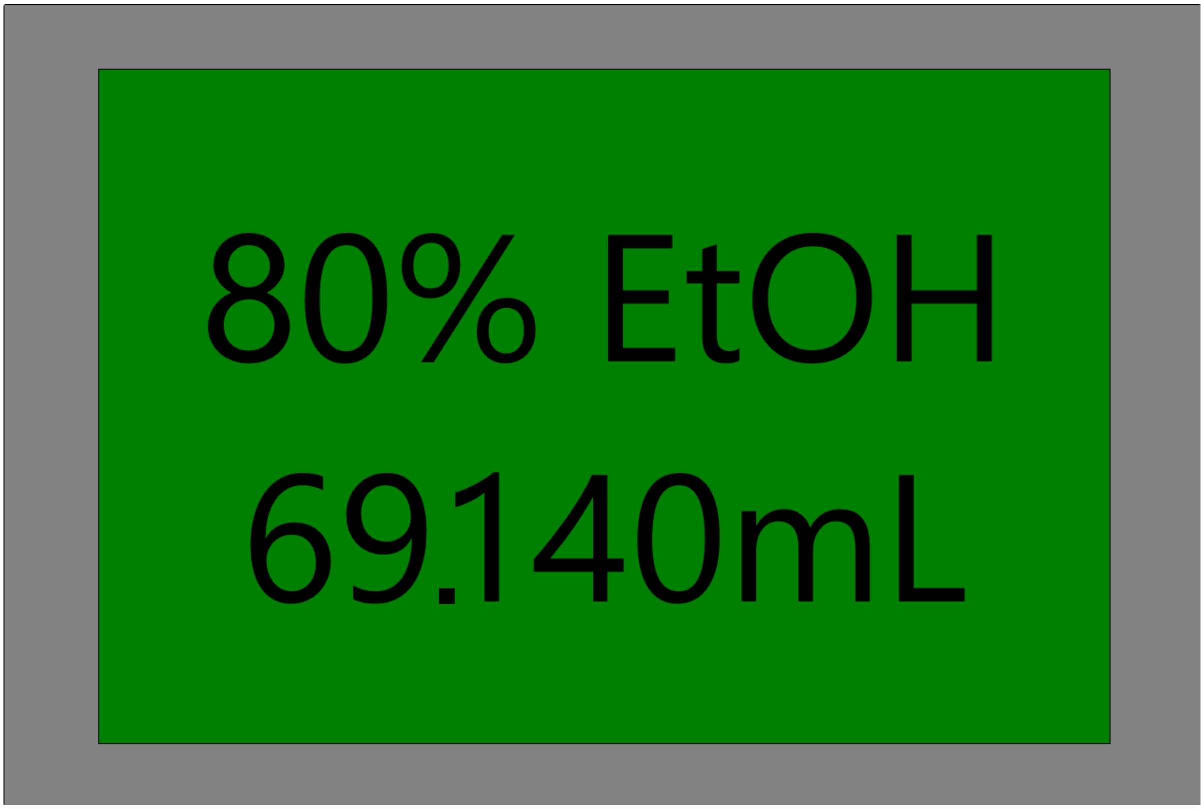

Place 80% EtOH(AgilentReservoir) on position P3

Labware Setup for Trypsin (ab_0932)

	1	2	3	4	5	6	7	8	9	10	11	12
A	Trypsin 650uL											
B	Trypsin 650uL											
C	Trypsin 650uL											
D	Trypsin 650uL											
E	Trypsin 650uL											
F	Trypsin 650uL											
G	Trypsin 650uL											
H	Trypsin 650uL											

Place Trypsin(ab_0932) on position P10

Labware Setup for Beads (ab_0932)

Notes:
Only need to put beads where samples are in the sample plate

	1	2	3	4	5	6	7	8	9	10	11	12
A	Beads 8 uL	Beads 8 uL	Beads 8 uL	Beads 8 uL	Beads 8 uL	Beads 8 uL	Beads 8 uL	Beads 8 uL	Beads 8 uL	Beads 8 uL	Beads 8 uL	Beads 8 uL
B	Beads 8 uL	Beads 8 uL	Beads 8 uL	Beads 8 uL	Beads 8 uL	Beads 8 uL	Beads 8 uL	Beads 8 uL	Beads 8 uL	Beads 8 uL	Beads 8 uL	Beads 8 uL
C	Beads 8 uL	Beads 8 uL	Beads 8 uL	Beads 8 uL	Beads 8 uL	Beads 8 uL	Beads 8 uL	Beads 8 uL	Beads 8 uL	Beads 8 uL	Beads 8 uL	Beads 8 uL
D	Beads 8 uL	Beads 8 uL	Beads 8 uL	Beads 8 uL	Beads 8 uL	Beads 8 uL	Beads 8 uL	Beads 8 uL	Beads 8 uL	Beads 8 uL	Beads 8 uL	Beads 8 uL
E	Beads 8 uL	Beads 8 uL	Beads 8 uL	Beads 8 uL	Beads 8 uL	Beads 8 uL	Beads 8 uL	Beads 8 uL	Beads 8 uL	Beads 8 uL	Beads 8 uL	Beads 8 uL
F	Beads 8 uL	Beads 8 uL	Beads 8 uL	Beads 8 uL	Beads 8 uL	Beads 8 uL	Beads 8 uL	Beads 8 uL	Beads 8 uL	Beads 8 uL	Beads 8 uL	Beads 8 uL
G	Beads 8 uL	Beads 8 uL	Beads 8 uL	Beads 8 uL	Beads 8 uL	Beads 8 uL	Beads 8 uL	Beads 8 uL	Beads 8 uL	Beads 8 uL	Beads 8 uL	Beads 8 uL
H	Beads 8 uL	Beads 8 uL	Beads 8 uL	Beads 8 uL	Beads 8 uL	Beads 8 uL	Beads 8 uL	Beads 8 uL	Beads 8 uL	Beads 8 uL	Beads 8 uL	Beads 8 uL

Place Beads(ab_0932) on position P7

New Labware Group Deck Setup

Ensure that labware is placed on the deck in the positions below.

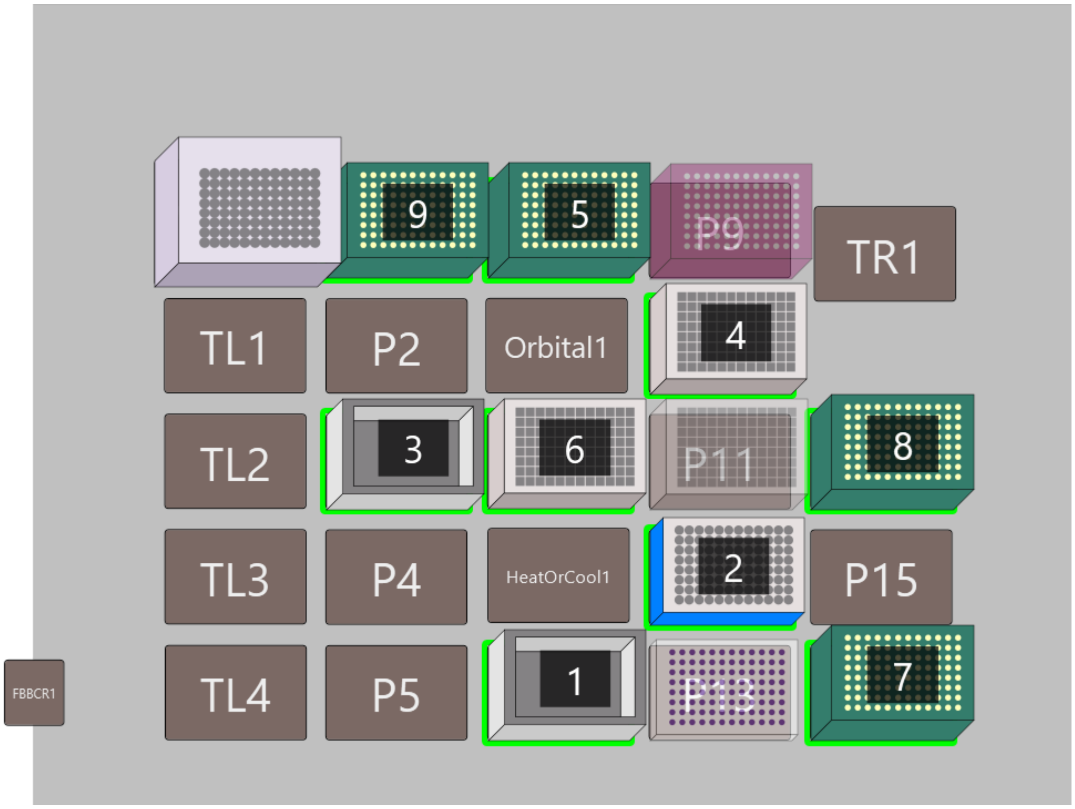

#	Name	Type	Position	Stack Depth
1	EtOH	AgilentReservoir	P8	1
2	Magnet	magnum_flx	P12	1
3	80% EtOH	AgilentReservoir	P3	1
4	Trypsin	ab_0932	P10	1
5	Trypsin_Tips	BC190F	P6	1
6	Beads	ab_0932	P7	1
7	Sample_Tips	BC190F	P16	1
8	EtOH_Tips	BC190F	P14	1
9	EtOH_Wash	BC190F	P1	1

Final Deck Setup

Below is the final deck layout. Ensure that all the highlighted labware are in the specified positions.

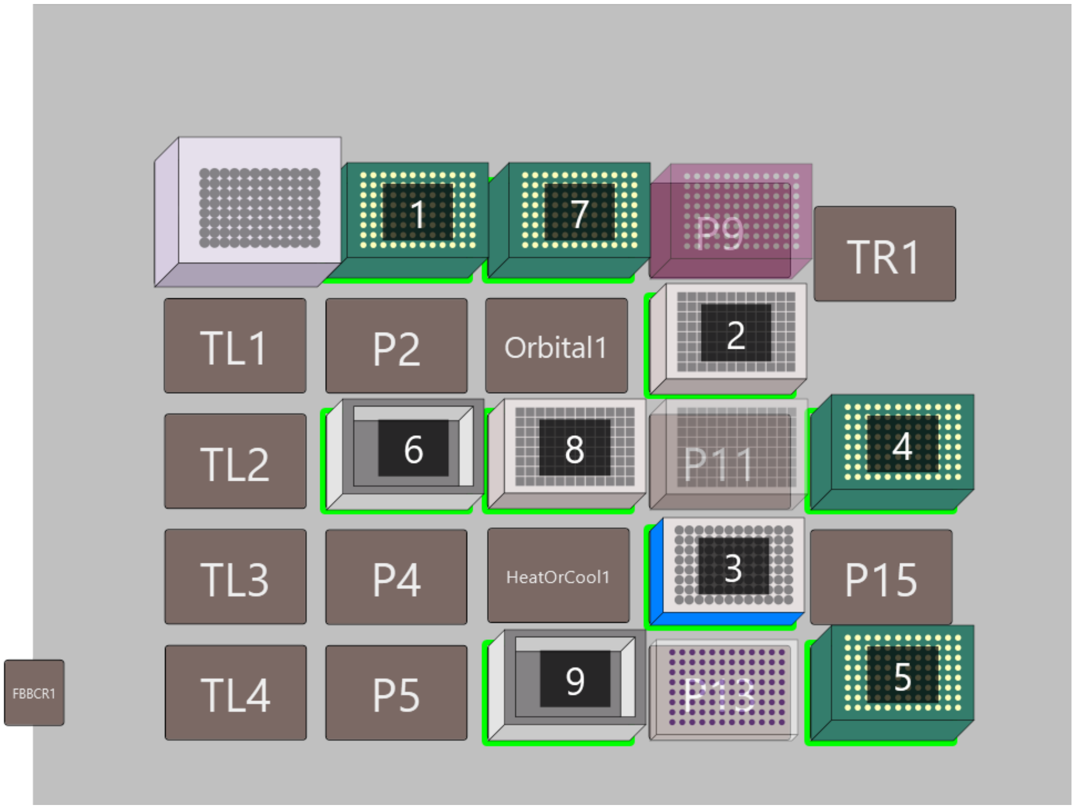

#	Name	Type	Position	Stack Depth
1	EtOH_Wash	BC190F	P1	1
2	Trypsin	ab_0932	P10	1
3	Magnet	magnum_flx	P12	1
4	EtOH_Tips	BC190F	P14	1
5	Sample_Tips	BC190F	P16	1
6	80% EtOH	AgilentReservoir	P3	1
7	Trypsin_Tips	BC190F	P6	1
8	Beads	ab_0932	P7	1
9	EtOH	AgilentReservoir	P8	1

Method Details

Labware Setup

This report shows where to place labware on the deck. The quantities and types of labware are listed below.
Start Time: (Print Time: 1/14/2025 1:16:17 PM)

Method: SP3 Bead_V1.1_Pauses_50ul, **Project:** Biomek i5 MC

Overall Setup Information

The left pod should have no tips loaded.

New Labware Group Deck Setup

Ensure that labware is placed on the deck in the positions below.

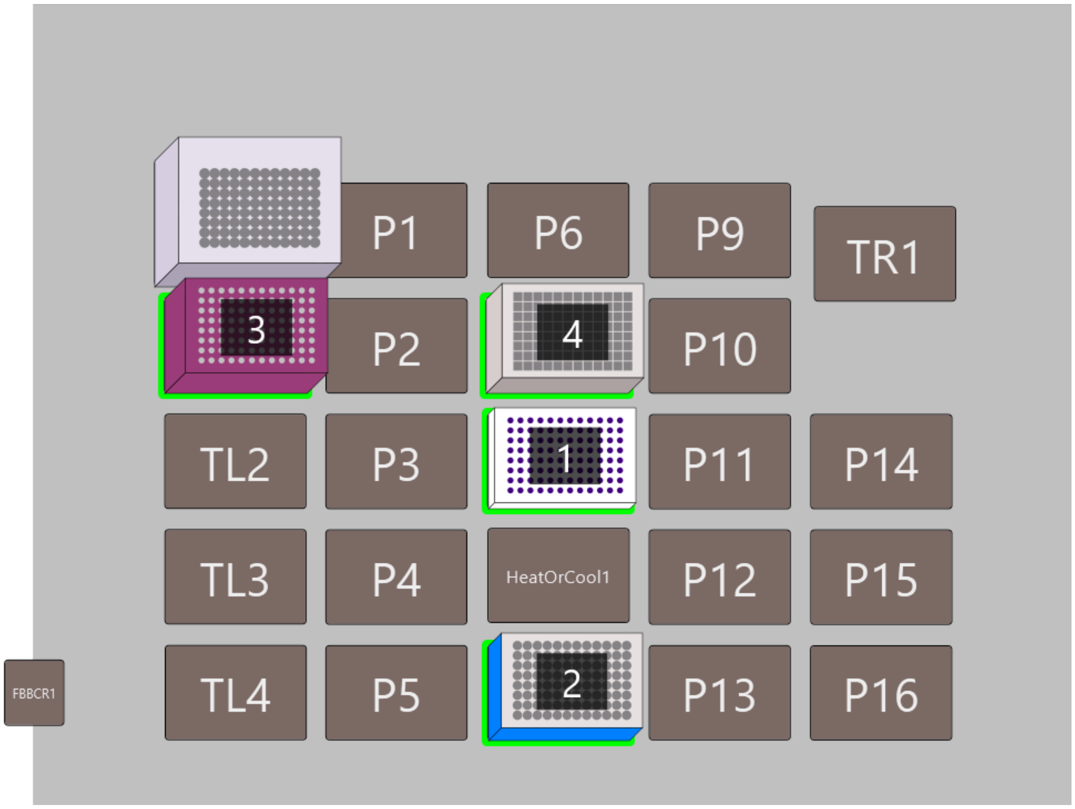

#	Name	Type	Position	Stack Depth
1	Supernatent	Bio_RadPCR96	P7	1
2	Magnet	magnum_flx	P8	1
3	Coll_Tips	BC90	TL1	1
4	Beads	ab_0932	Orbital1	1

Final Deck Setup

Below is the final deck layout. Ensure that all the highlighted labware are in the specified positions.

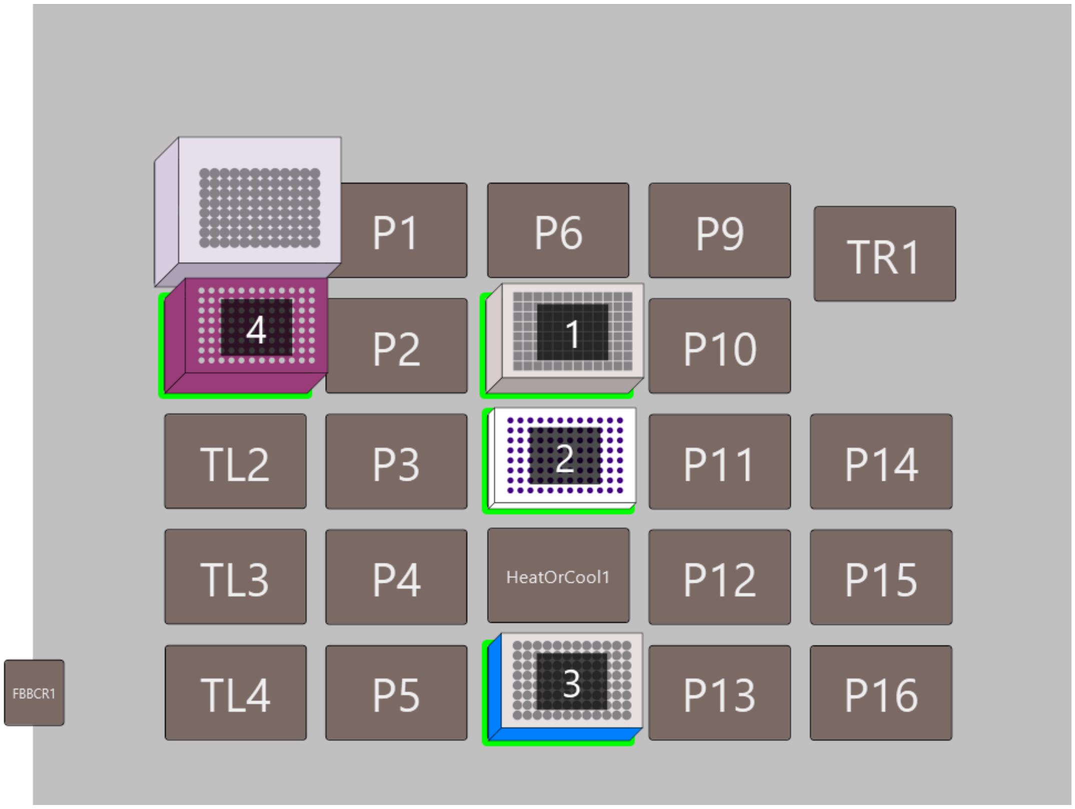

#	Name	Type	Position	Stack Depth
1	Beads	ab_0932	Orbital1	1
2	Supernatent	Bio_RadPCR96	P7	1
3	Magnet	magnum_flx	P8	1
4	Coll_Tips	BC90	TL1	1

Method Details

Labware Setup

This report shows where to place labware on the deck. The quantities and types of labware are listed below.
Start Time: (Print Time: 1/14/2025 1:24:44 PM)

Method: TMT Labeling_V1.1, **Project:** Biomek i5 MC

Overall Setup Information

The left pod should have no tips loaded.

1. Tips and Empty Labware

Labware Setup for Final Tubes (BCTubeBlock_2mlTubes)

Notes:
Add 1 tube for each experiment you are running.

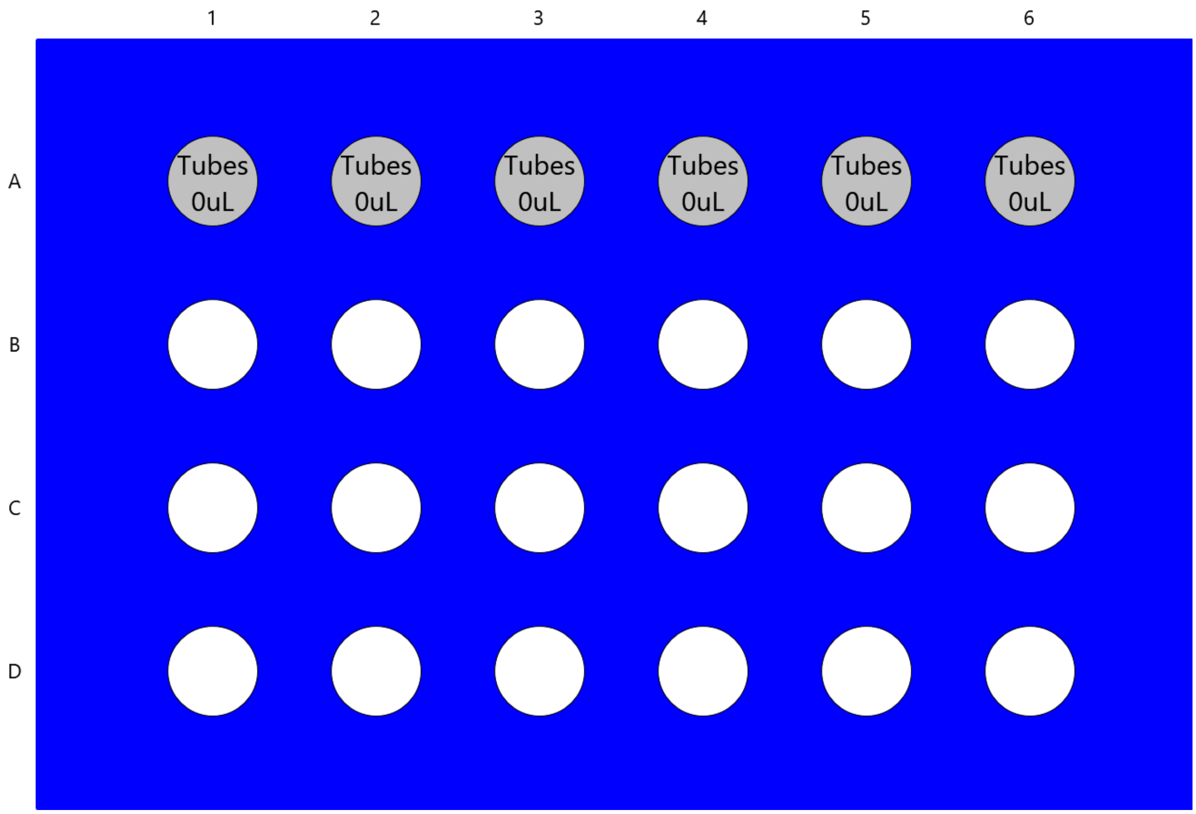

Place Final Tubes(BCTubeBlock_2mlTubes) on position P1

Tips and Empty Labware Deck Setup

Ensure that labware is placed on the deck in the positions below.

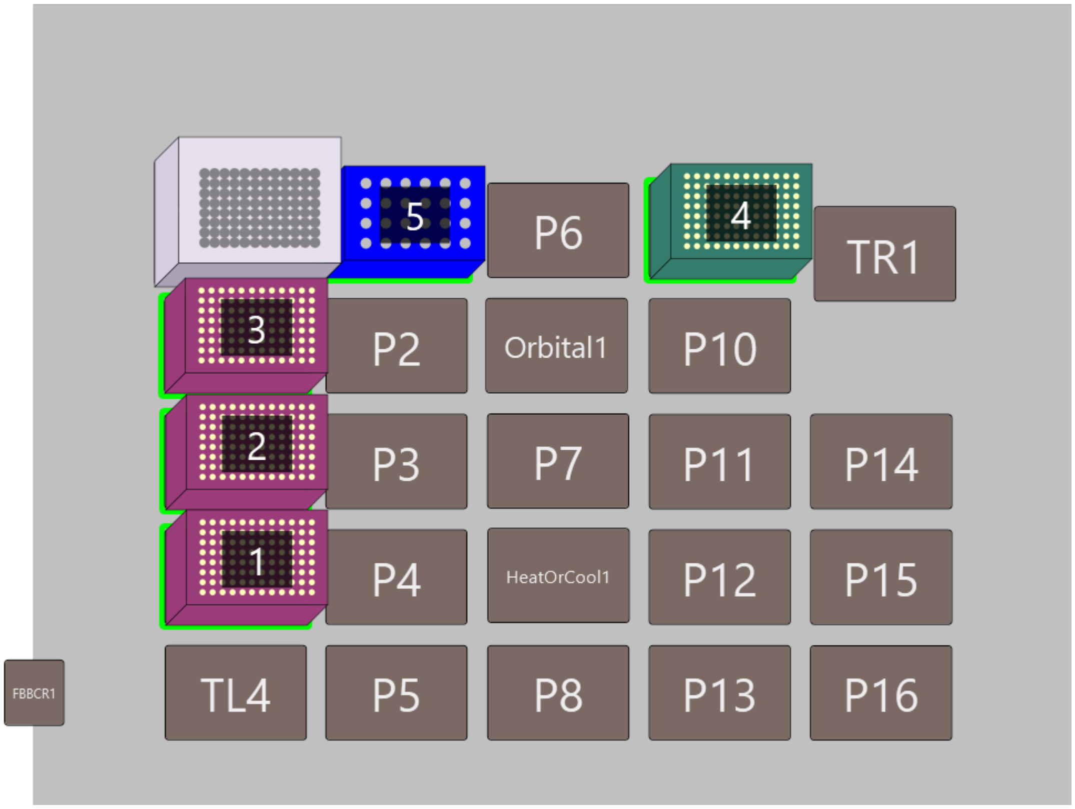

#	Name	Type	Position	Stack Depth
1	AmBi Tips	BC50F	TL3	1
2	TMT Tips	BC50F	TL2	1
3	Water Tips	BC50F	TL1	1
4	Pooling Tips	BC190F	P9	1
5	Final Tubes	BCTubeBlock_2mlTubes	P1	1

2. Reagents

Labware Setup for Reagents (ab_0932)

Notes:
Master Mix Composition for 1 reactions:
10uL Hepes + 32.5uL H20uL + .5uL TCEP

	1	2	3	4	5	6	7	8	9	10	11	12
A			Water 145uL	AmBi 73uL	FAcid 611.6uL							
B			Water 145uL	AmBi 73uL								
C			Water 145uL	AmBi 73uL								
D			Water 145uL	AmBi 73uL								
E			Water 145uL	AmBi 73uL								
F			Water 145uL	AmBi 73uL								
G			Water 145uL	AmBi 73uL								
H			Water 145uL	AmBi 73uL								

Place Reagents(ab_0932) on position P8

Labware Setup for LVLTubes (LVL_MX500_TMT_Tubes)

	1	2	3	4	5	6	7	8	9	10	11	12
A	Trypsin 121uL	S1 265uL	S9 265uL									
B		S2 265uL	S10 265uL									
C		S3 265uL	S11 265uL									
D		S4 265uL	S12 265uL									
E		S5 265uL	S13 265uL									
F		S6 265uL	S14 265uL									
G		S7 265uL	S15 265uL									
H		S8 265uL	S16 265uL									

Place LVLTubes(LVL_MX500_TMT_Tubes) on position P4

Labware Setup for Sample (Bio_RadPCR96)

Notes:
Arrange each Experiment in the layout shown Below.

Up to 6 Experiments can fit on one 96 Well Plate

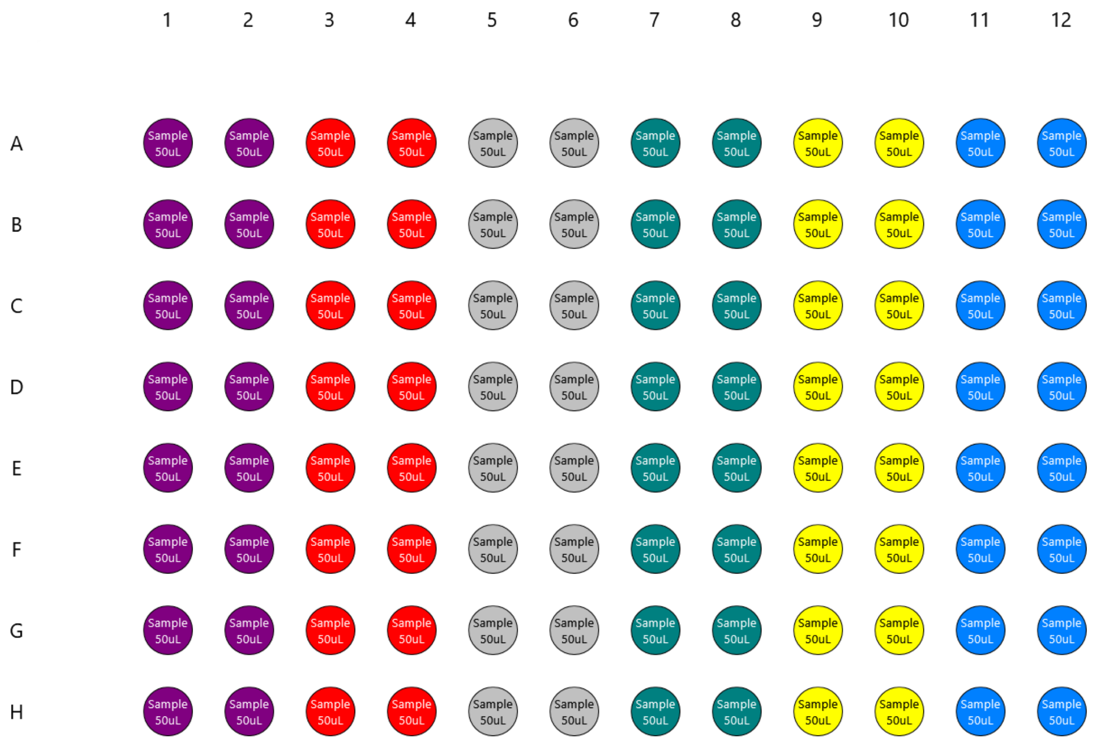

Place Sample(Bio_RadPCR96) on position P7

Reagents Deck Setup

Ensure that labware is placed on the deck in the positions below.

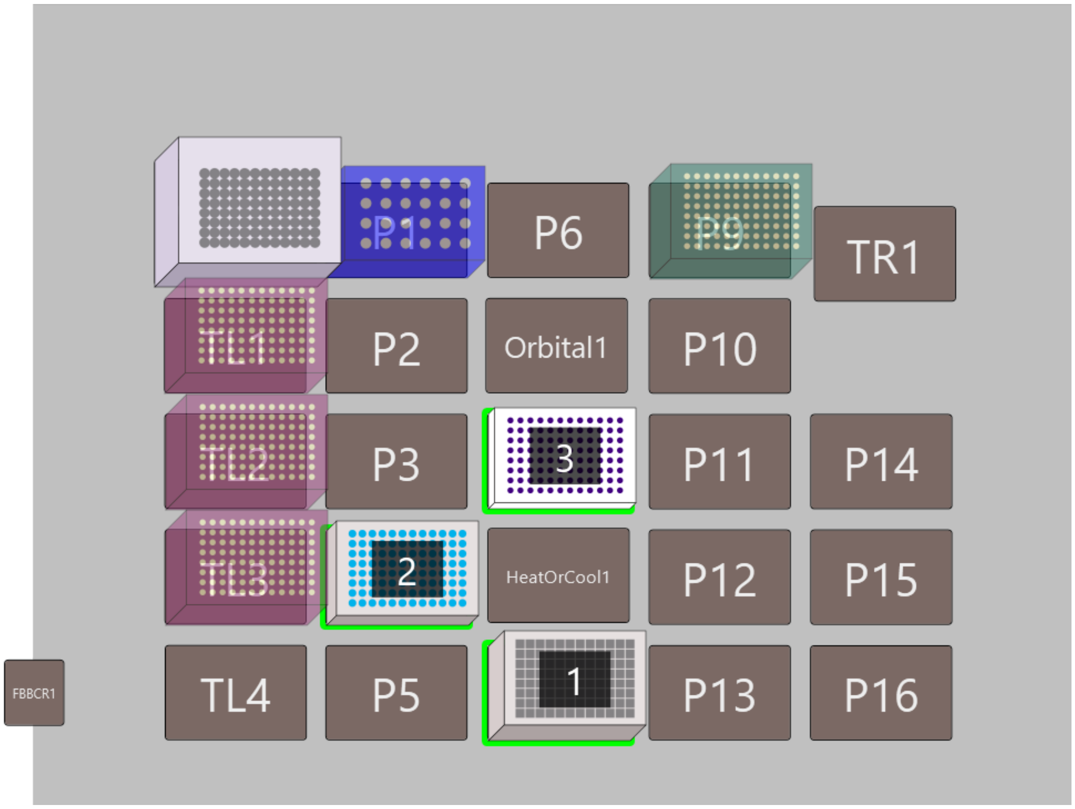

#	Name	Type	Position	Stack Depth
1	Reagents	ab_0932	P8	1
2	LVLTubes	LVL_MX500_TMT_Tubes	P4	1
3	Sample	Bio_RadPCR96	P7	1

Final Deck Setup

Below is the final deck layout. Ensure that all the highlighted labware are in the specified positions.

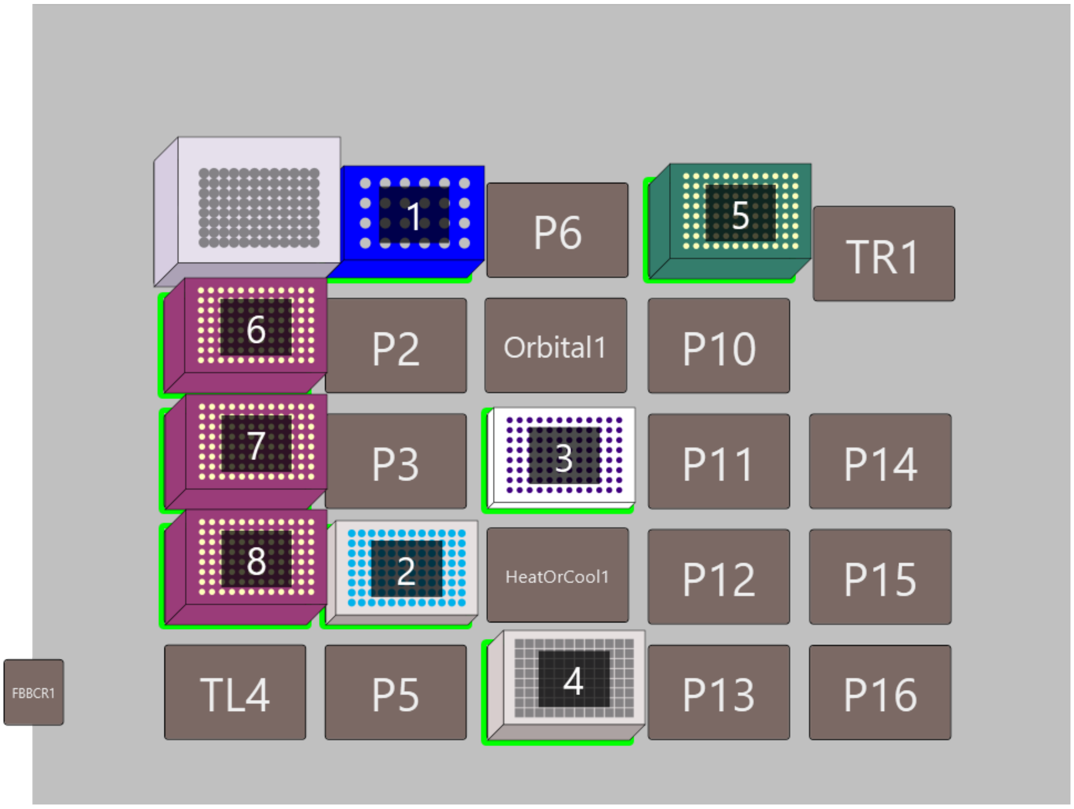

#	Name	Type	Position	Stack Depth
1	Final Tubes	BCTubeBlock_2mlTubes	P1	1
2	LVLTubes	LVL_MX500_TMT_Tubes	P4	1
3	Sample	Bio_RadPCR96	P7	1
4	Reagents	ab_0932	P8	1
5	Pooling Tips	BC190F	P9	1
6	Water Tips	BC50F	TL1	1
7	TMT Tips	BC50F	TL2	1
8	AmBi Tips	BC50F	TL3	1

Method Details
